# Single-crystal organometallic perovskite optical fibers

**DOI:** 10.1126/sciadv.abq8629

**Published:** 2022-09-23

**Authors:** Yongfeng Zhou, Michael A. Parkes, Jinshuai Zhang, Yufei Wang, Michael Ruddlesden, Helen H. Fielding, Lei Su

**Affiliations:** ^1^ School of Engineering and Materials Science, Queen Mary University of London, London E1 4NS, UK.; ^2^ Department of Chemistry, University College London, 20 Gordon Street, London WC1H 0AJ, UK.

## Abstract

Semiconductors in their optical-fiber forms are desirable. Single-crystal organometallic halide perovskites have attractive optoelectronic properties and therefore are suitable fiber-optic platforms. However, single-crystal organometallic perovskite optical fibers have not been reported before due to the challenge of one-directional single-crystal growth in solution. Here, we report a solution-processed approach to continuously grow single-crystal organometallic perovskite optical fibers with controllable diameters and lengths. For single-crystal MAPbBr_3_ (MA = CH_3_NH_3_^+^) perovskite optical fiber made using our method, it demonstrates low transmission losses (<0.7 dB/cm), mechanical flexibilities (a bending radius down to 3.5 mm), and mechanical deformation–tunable photoluminescence in organometallic perovskites. Moreover, the light confinement provided by our organometallic perovskite optical fibers leads to three-photon absorption (3PA), in contrast with 2PA in bulk single crystals under the same experimental conditions. The single-crystal organometallic perovskite optical fibers have the potential in future optoelectronic applications.

## Teaser

A single-crystal halide perovskite optical fiber with suitable optoelectronic performances can be grown in solution.

## INTRODUCTION

Organometallic halide perovskites are attractive candidates for a wide range of applications in photovoltaic and optoelectronic devices due to their simple solution preparations (*1*), rich chemical and structural diversities (*2*), large carrier mobilities (*3*), and tunable bandgaps (*4*). The optoelectronic applications of organometallic hybrid perovskites have been demonstrated in high-efficiency solar cells (*5*), light-emitting diodes (*6*), photodetectors (*7*), and lasers (*8*). More recently, increasing research evidence indicates their promising third-order nonlinear optical properties (*9*, *10*), piezoelectric response (*11*), and thermoelectric properties (*12*). In particular, single-crystal organometallic perovskites have superior performances compared to their polycrystalline counterparts, such as high stability, low optical transmission loss, long charge-carrier lifetime, and long carrier diffusion length as a result of low defect densities (*13*). Several fabrication methods have then been reported for single-crystal organometallic perovskites, including temperature-lowering crystallization (*14*), antisolvent vapor-assisted crystallization (*13*), liquid-diffused separation-induced crystallization (*15*), and inverse temperature crystallization (*16*).

Semiconductor core fibers not only have a wide range of applications in optics, as sources (*17*), detectors (*18*), and nonlinear response media (*19*), but also emerge as versatile platforms of advanced functional fibers for multifunctional smart fabric in sensors (*20*), photovoltaics (*21*), thermoelectrics (*22*), piezoelectrics (*23*), p-n diodes (*24*), etc. Several types of semiconductor materials have been used successfully to make optical fibers, including silicon (*25*), germanium (*26*), III-V compounds (*27*), II-VI compounds (*28*), and chalcogenides (*20*). Their fabrication approaches are based primarily on thermal drawing (*25*), pressure-assisted physical filling (*29*), and high-pressure chemical vapor deposition (*26*). Single-crystal silicon and germanium optical fibers were achieved through additional laser recrystallization on polycrystalline fibers (*30*–*32*).

In particular, single-crystal organometallic perovskite optical fibers have many advantages in high-speed all-fiber optoelectronics. Organometallic perovskites have direct bandgap and low defect densities, which are efficient at emitting light (*33*, *34*). Direct bandgap low-loss single-crystal organometallic perovskite optical fibers can be a suitable candidate for the integration of the light source into all-fiber optical networks. Moreover, the bandgap of single-crystal organometallic perovskite optical fibers can be continuously engineered by simply changing the elemental composition through the chemical precursors (fig. S1). In addition, the reported optoelectronic and nonlinear optical properties (*3*, *7*, *9*, *10*) suggest that single-crystal organometallic perovskite optical fibers are promising platforms for detectors and nonlinear optics. By considering all these properties mentioned above, single-crystal organometallic perovskite optical fibers could be an all-around candidate in high-speed all-fiber optoelectronics, where light can be generated, modulated, and detected within an optical fiber.

Despite the potential of single-crystal organometallic perovskite materials in optoelectronic and electronic devices, single-crystal organometallic perovskite optical fibers are beyond the state of the art. It is challenging to achieve the continuous one-directional growth of the single-crystal organometallic perovskites in an axial direction, while limiting its growth in the radial direction and inhibiting random nucleation (*35*). Wafer-scale single-crystal organometallic perovskite thin film that realizes planar growth with precise control of thickness was achieved by a lithography-assisted epitaxial inverse temperature growth method (*36*). Organometallic perovskite polycrystalline and monocrystalline microwires synthesized in solution have been reported (*37*, *38*); however, their lengths were on the micrometer scale and their geometric dimensions were not controllable. Template solution growth methods using polydimethylsiloxane groove templates and capillaries have been developed to produce organometallic perovskite nanowires and microwires for laser applications (*39*, *40*). Although the cross-section dimensions of these nanowires or microwires were controlled to a certain extent, they were polycrystalline perovskites, and their lengths were also only on the micrometer scale.

## RESULTS

### Fabrication of single-crystal organometallic perovskite optical fibers

**Fig. 1.**
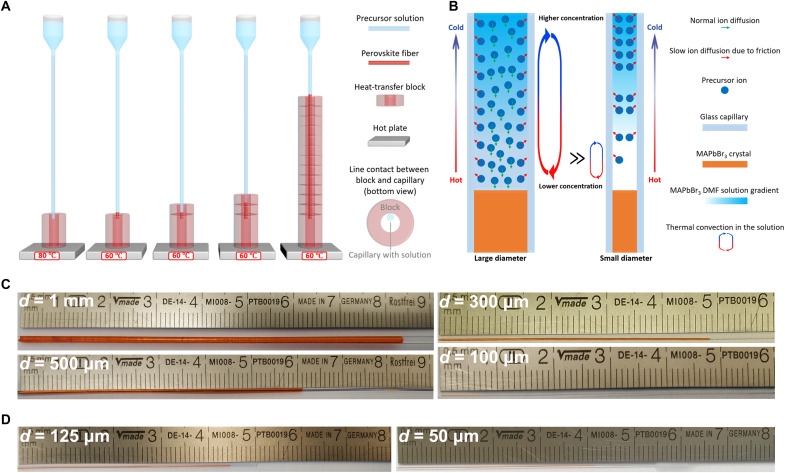
The solution-processed, space-confined, inverse temperature crystallization method to fabricate single-crystal organometallic perovskite optical fibers. (**A**) The schematic diagram of the single-crystal organometallic perovskite optical fiber fabrication method. (**B**) Schematic illustrations of the ion diffusion rate in the capillaries with different inner diameters, *d*. (**C**) Single-crystal MAPbBr_3_ perovskite optical fibers of different lengths and diameters with silica cladding. (**D**) Single-crystal MAPbBr_3_ perovskite optical fibers of different lengths and diameters with PTFE cladding.

First, we propose a solution-processed, space-confined, inverse temperature crystallization method for the growth of single-crystal organometallic perovskite optical fibers continuously along the axial direction, as shown in Fig. 1A. The detailed method description can be found in Materials and Methods. Capillaries are used to realize the axial directional growth while limiting the growth in the radial directions. The gradually changed heating position, line contact, and temperature changes during the process ensure continuous growth while preventing random nucleation in the axial direction. The length of the fiber can be controlled, and the cross section of the perovskite fiber core can be varied according to the inner diameters of the capillary. This method is applicable to other organometallic perovskite materials that can be processed in solution. The cladding material can be glass or other materials.

Figure 1C shows single-crystal MAPbBr_3_ perovskite optical fibers of different lengths and diameters fabricated with our method by using silica capillaries. For 100-μm-core-diameter single-crystal organometallic perovskite optical fibers with silica cladding, the lengths we currently can achieve are limited to several millimeters (e.g., the 100-μm-core-diameter 7-mm-long fiber in Fig. 1C). The length of the fibers with larger diameters (such as 300 μm, 500 μm, and 1 mm in our experiments) can grow indefinitely in theory. We find that the smaller the inner diameter of the glass capillary is, the longer it takes for the organometallic perovskite fiber to grow to a certain length. For glass capillaries with an inner core diameter smaller than 300 μm, the growth rate usually decreases gradually during the crystallization growth process until the growth finally stops.

We believe that this growth dependence on the capillary diameter is due to the insufficient long-range transport of precursor ions along capillaries; i.e., the depleted precursors within capillaries of smaller diameters cannot be sufficiently replenished in real time for continuous crystal growth. We consider that the diffusion rate of precursor ions dissolved in the organic solvent is determined by the transport speed of solvent molecules along the capillary axial direction, which is influenced by two main mechanisms in our system. First, the wetting glass capillary surface with high surface energy normally attracts solvent in precursor solution, which plays a negative role for ion diffusion (*41*, *42*). The glass capillaries with their large attraction force to solvent molecules drag down the speed of solvent molecule transport (*43*). Second, in contrast to the attraction force that slows down the solvent transport, thermal convection in the solution caused by temperature gradients facilitates the precursor ion diffusion. When the capillary diameter is large enough, even though the solution transport near the capillary surface is dragged down by the attraction force, effective long-range thermal convection can still be achieved within the capillary for the precursor solution farther away from the inner glass capillary surface, as shown in the large-diameter capillary in Fig. 1B. When the capillary diameter is too small (i.e., <300 μm), the attraction force induced by the inner glass capillary surface affects most of the solvent molecules within the capillary and drags down the speed of the solution transport (see the small-diameter capillary in Fig. 1B); therefore, the thermal convection works only over a short distance, which results in insufficient ion diffusion to replenish the depleted precursor ions.

This growth limitation could be improved by using nonwetting cladding materials or introducing a nonwetting film on the inner surface of the capillary, which could reduce or eliminate the attraction force to solvent molecules. Nonwetting polytetrafluoroethylene (PTFE) was chosen for the cladding material to overcome the growth limitation of our method. Figure 1D illustrates 50- and 125-μm-core-diameter single-crystal MAPbBr_3_ perovskite optical fibers with lengths of several centimeters fabricated with our method by using PTFE cladding. Organometallic perovskite optical fibers with even smaller core diameters and longer lengths can also be achieved through these modifications. Our method provides a solution to break the current aspect ratio limit for single-crystal perovskite in-solution growth (*7*, *44*).

### Material characterization of single-crystal organometallic perovskite optical fibers

**Fig. 2.**
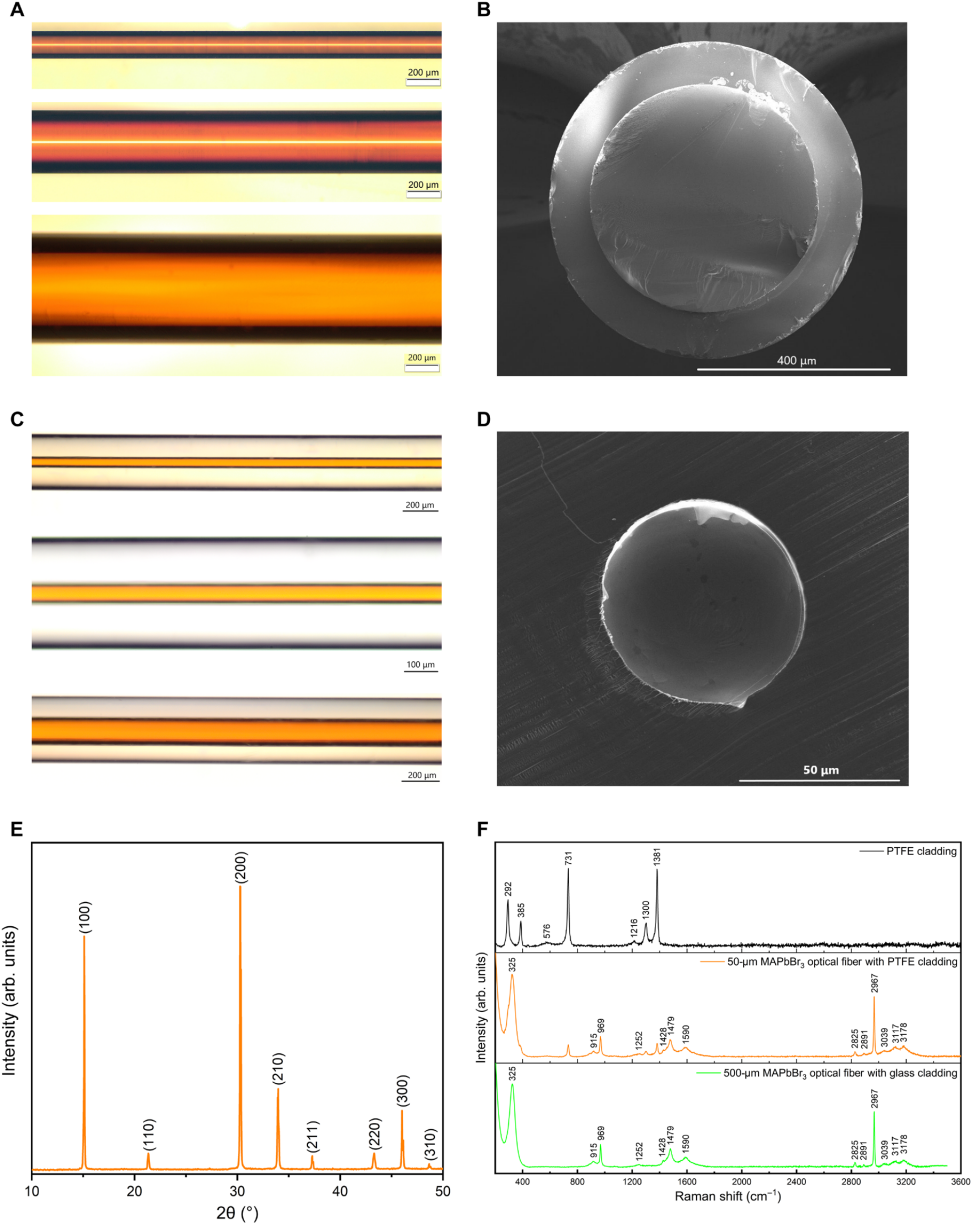
Material characterization of single-crystal organometallic perovskite optical fibers. (**A**) Optical microscopy images of MAPbBr_3_ perovskite fibers with silica cladding. (**B**) SEM image of the 500-μm-core-diameter fiber cross section with silica cladding. (**C**) Optical microscopy images of MAPbBr_3_ perovskite optical fibers with PTFE cladding. (**D**) SEM image of the 50-μm-core-diameter fiber cross section with PTFE cladding. (**E**) Powder XRD of ground MAPbBr_3_ crystals from a 500-μm perovskite core. (**F**) Raman spectra of MAPbBr_3_ fibers with core diameters of 50 and 500 μm.

Next, we characterized our single-crystal organometallic perovskite optical fibers. The optical microscopy images (Fig. 2, A and C) of MAPbBr_3_ single-crystal perovskite optical fibers display high-quality perovskite cores wrapped by the glass cladding and PTFE cladding. Figure S1 also illustrates the optical microscopy images of MAPbBr_2.5_Cl_0.5_ and MAPbCl_3_ single-crystal perovskite optical fibers. The optical microscopy images of MAPbBr_3_ polycrystalline fibers are shown in fig. S2 as a comparison. The scanning electron microscope (SEM) images (Fig. 2, B and D, and fig. S4) of the fiber cross sections reveal no interfacial irregularities at the core/cladding boundary and no visible grain boundaries and voids on the cross-sectional areas. High-resolution SEM images are also collected for the cross sections of the single-crystal organometallic perovskite optical fibers, where no grain structures are observed (fig. S3). Energy-dispersive x-ray spectroscopy (EDX) analysis (fig. S4) shows uniform distribution of chemical compositions within the organometallic perovskite core.

The phase purity of the as-grown MAPbBr_3_ single-crystal perovskite optical fibers is confirmed by x-ray diffraction (XRD) performed on powders ground from a 500-μm perovskite core (Fig. 2E). Raman spectroscopy analyses of the 50- and 500-μm MAPbBr_3_ fibers are conducted at an excitation wavelength of 633 nm to avoid the fluorescence background, as shown in Fig. 2F. The Raman shifts of vibration modes and peak assignments for this organometallic perovskite optical fiber are summarized in table S1. The measured results are in good agreement with results obtained for single-crystal MAPbBr_3_ perovskites (*45*), thereby implying a high degree of phase purity and crystallinity of our single-crystal organometallic perovskite fibers.

**Fig. 3.**
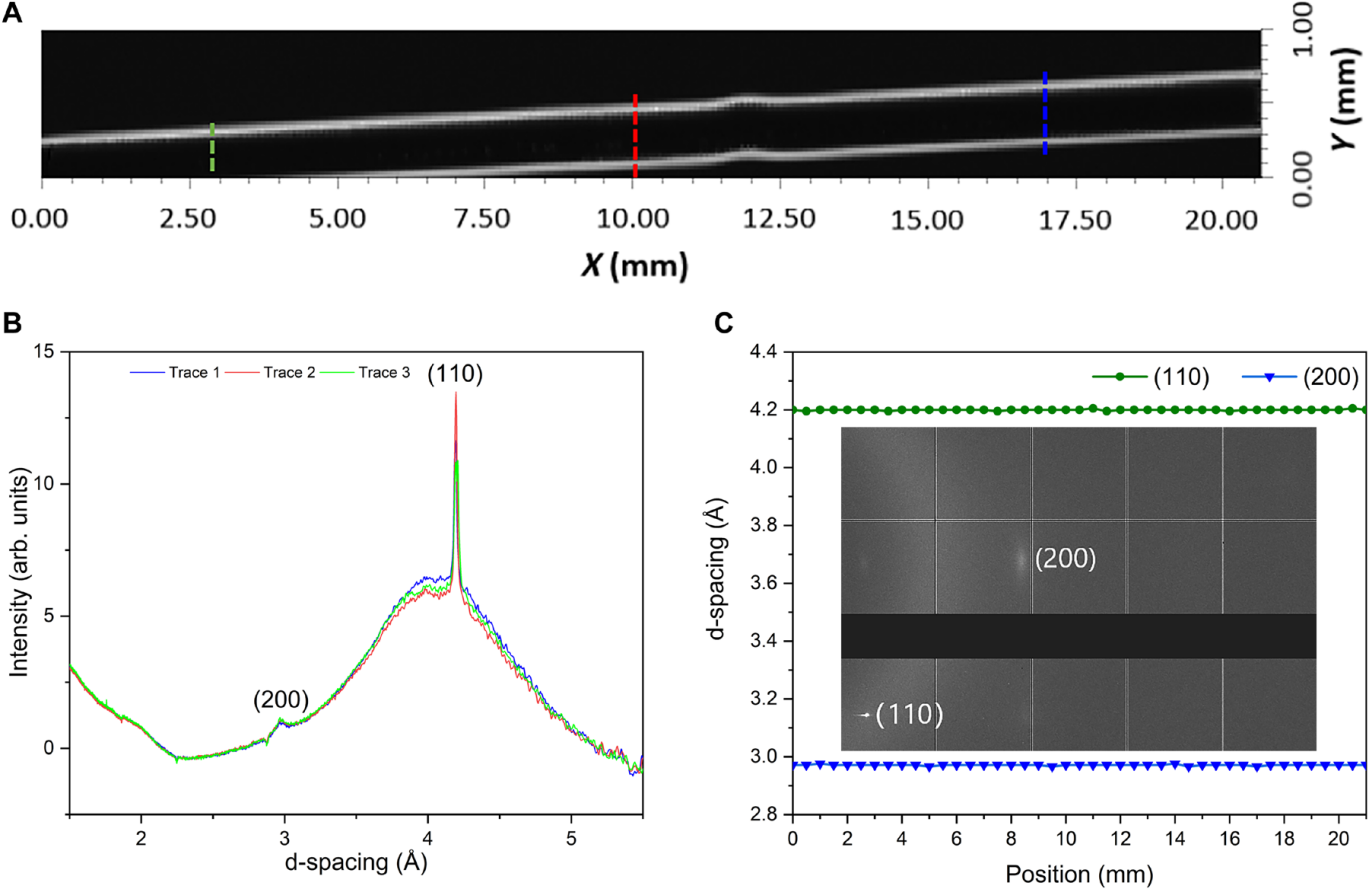
Synchrotron XRD analysis of single-crystal organometallic perovskite optical fibers. (**A**) The intensity map of the (110) peak in the whole organometallic perovskite fiber range through synchrotron XRD analysis. (**B**) d-spacing distributions and corresponding intensities of three different positions [as shown in (A)] in the organometallic perovskite fiber. (**C**) Measured lattice spacings from synchrotron XRD as a function of the position along the fiber and the diffraction pattern. Inset: Diffraction pattern of the 300-μm single-crystal MAPbBr_3_ fiber.

The single-crystal feature of the organometallic perovskite optical fiber is further investigated by synchrotron XRD in transmission mode. For the 300-μm MAPbBr_3_ fiber tested, owing to the transmission mode measurement and the cylindrical shape of the fiber, the transmitted x-ray attenuation due to the Pb element in MAPbBr_3_ is lowest at the edges (thinnest perovskite material), gradually increases toward the center of the fiber, and peaks along the center line (thickest perovskite material). A dominated diffraction spot of (110) and an associated weak spot of (200) are observed in the diffraction pattern, as shown in the inset of Fig. 3C. The dominant peak (110) is observed in the entire fiber, while the (200) peak is not obvious in the middle region of the fiber (the thicker part) due to higher attenuation, as shown in fig. S5. Figure 3A is the synchrotron transmission intensity map of the (110) peak along the entire single-crystal organometallic perovskite optical fiber, where the edges of the fiber in the map (thinner perovskite material) look brighter compared to the middle region of the fiber. The gradient of the (110) peak intensity from the edge to the center can be seen in fig. S5. The d-spacing distributions and corresponding intensities of three different positions in the single-crystal organometallic perovskite fiber are almost the same (Fig. 3B). Figure 3C shows the measured lattice spacings from XRD as a function of the position along the fiber. Analyses are conducted on the raw data of XRD by using Dawn Diamond software (*30*). All these suggest that the orientations and d-spacings associated with the (110) and (200) peaks are maintained over the entire 300-μm core and 2.1-cm length of the organometallic perovskite fiber and proves its single-crystal character. Moreover, the flexible 50-μm MAPbBr_3_ fiber with PTFE cladding is also tested by synchrotron XRD; fig. S6 illustrates the synchrotron XRD analysis of a flexible fiber. The diffraction spots instead of rings suggest the single-crystal feature of this fiber; however, the gradually changing lattice orientations along the axis direction in this flexible fiber are different from that of the rigid fiber above (Fig. 3). A possible explanation for the gradually changed lattice orientations is the twists or bends present in the flexible fiber. Compared with the rigid glass capillaries, the PTFE capillaries are flexible and can be easily deformed. As a result, twists or bends are introduced to the flexible fiber when we attach the two ends of the fiber to a frame for the test or even during the crystal growth process. These may cause the fiber-core lattice orientation variations on the axial direction.

### Optical properties and photoelectric performances

**Fig. 4.**
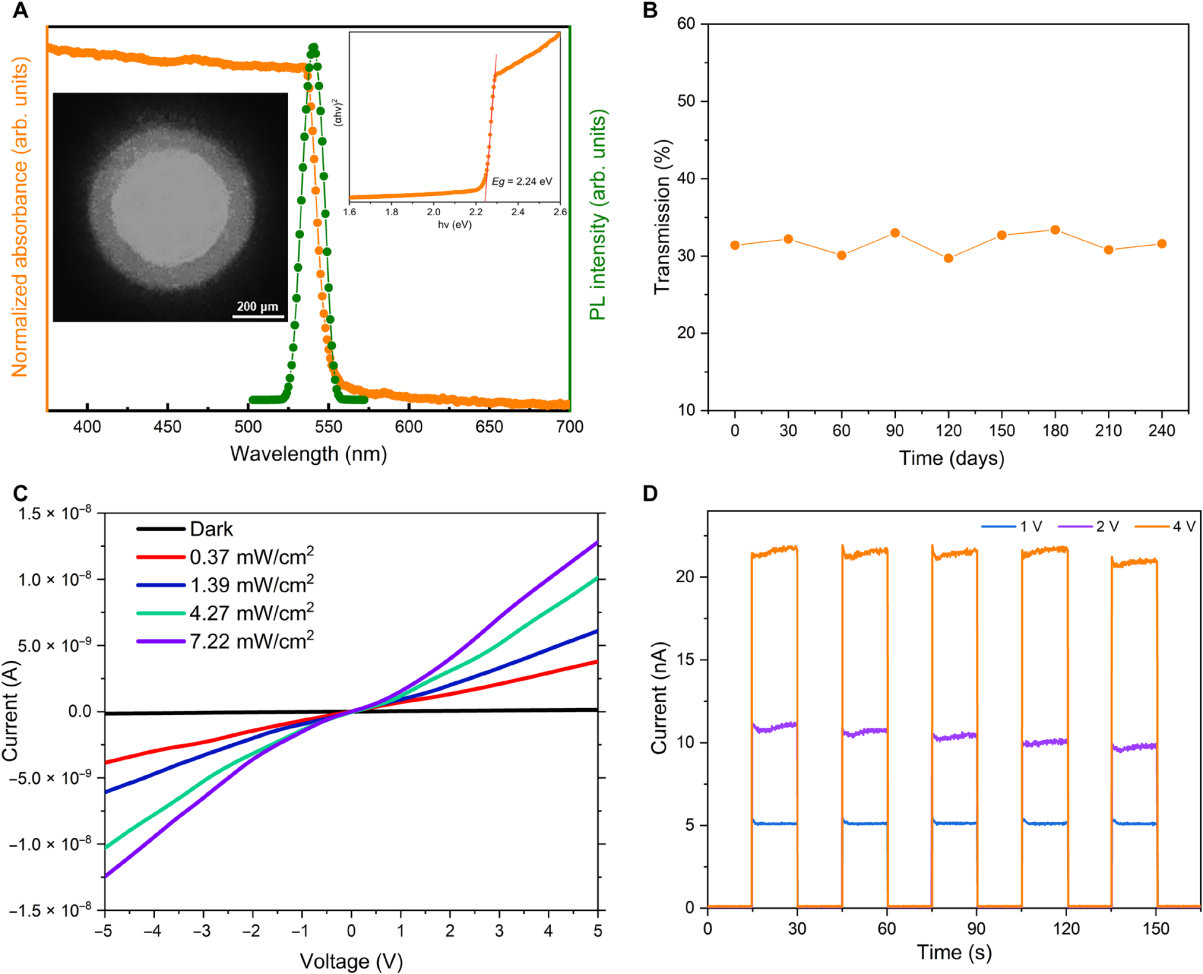
Optical properties and photoelectric performance of the single-crystal MAPbBr_3_ optical fiber. (**A**) Steady-state absorbance and photoluminescence of 500-μm MAPbBr_3_ optical fibers. Left inset: Near-field image captured by a CCD camera at the 500-μm fiber output. Right inset: the Tauc plot and the calculated optical bandgap (2.24 eV). (**B**) The transmission of a 500-μm-core-diameter, 31-mm-long single-crystal MAPbBr_3_ perovskite optical fiber in air over 240 days. (**C**) *I*-*V* curves of the 500-μm-core-diameter, 1-cm-long fiber in the dark and under 532 nm with different light irradiation intensities. (**D**) Dynamic photoresponse of the 500-μm-core-diameter, 1-cm-long fiber under a solar simulator (light intensity, 100 mW cm^−2^) with different bias voltages.

Further, we studied the optical properties of the single-crystal MAPbBr_3_ perovskite optical fiber. As shown in Fig. 4A, the single-crystal organometallic perovskite fiber material exhibits a sharp absorption edge, and the bandgap extracted from a Tauc plot is 2.24 eV. The single-crystal organometallic perovskite fiber material also shows a narrow photoluminescence (PL) peak at 540 nm. These findings are in line with the results reported in previous work concerning MAPbBr_3_ single crystals (*16*, *46*). It is worth mentioning that the bandgap of our fiber can be engineered simply by adjusting the halogens in the precursor solution (fig. S1). The optical transmission losses of the fibers are measured using the standard cutback technique. The average optical loss coefficients of the 300- and 500-μm optical fibers are 1.33 and 1.20 dB cm^−1^ at 785 nm, respectively. The smallest losses measured are 0.82 and 0.67 dB cm^−1^ for 300- and 500-μm optical fibers, respectively, as shown in fig. S7. These values provide experimentally measured propagation losses of organometallic halide perovskite waveguides (i.e., optical fibers). The origin of the transmission loss in our single-crystal optical fibers could be mainly due to those defects formed during the crystal growth. There are a few possible reasons for defect formation, namely, high-temperature growth, temperature variations, and capillary surface influence. First, the relatively high temperature employed in our inverse temperature crystallization method could be detrimental to crystal quality. It was recently reported that single-crystalline MAPbBr_3_ fabricated by liquid-diffused separation-induced crystallization at room temperature had 10 times higher PL intensity, more than 4 times longer carrier lifetime, and much smaller trap density compared to samples grown by inverse temperature crystallization (ITC) (*15*). Second, small variations in temperature may lead to notable variation in precursor solubility and influence crystal quality (*47*). Third, the capillary surface may cause defects near the crystal surface with the depth of a few tens of micrometers due to insufficient ion delivery and mismatch strain (*48*). The solution transport near the capillary surface is dragged down by the attraction force due to the wetting glass capillary surface, and insufficient ion delivery to the crystal surface would create a deficiency for one type of ions, or misfit defects. Defects could also be generated because of the strain imposed by the mismatch of the capillaries and the crystals during growth. To overcome these factors and to further reduce the transmission loss, there are a few approaches that can be used in the future, including lowering the fabrication temperature, minimizing temperature variations, and introducing a nonwetting film on the inner surface of capillary. The inset of Fig. 4A is the near-field image captured by a charge-coupled device (CCD) camera at the 500-μm fiber output with a 785-nm continuous-wave laser input, where well-confined guided light within the organometallic perovskite optical fiber core can be seen clearly. The steady-state absorption of single-crystal MAPbBr_3_ optical fibers remains relatively low for wavelengths longer than 550 nm (fig. S8), suggesting potential applications in the visible and infrared wavelength regions. The optical properties of our single-crystal organometallic perovskite optical fiber are found to be stable over a long period of time, owing to its single-crystal nature and the protection provided by the glass cladding. The optical fiber transmission loss remains largely unchanged after being stored for 8 months under ambient laboratory conditions (Fig. 4B). Since the fiber end facets are exposed directly to ambient air, they require polishing before each measurement. The small fluctuations among the optical loss measurements shown in Fig. 4B are due to measurement errors and differences introduced by polishing before each measurement.

Moreover, the photoelectric performance is studied by using a single-crystal MAPbBr_3_ fiber photodetector (500-μm-core-diameter, 1-cm-long fiber). Figure 4C shows the optoelectronic current of the fiber under illumination with 532-nm light at different intensities. These photocurrent values are much larger than those of a polycrystalline MAPbBr_3_ milliwire photodetector with similar length due to the superior properties of the single crystal (*49*). A stable dynamic photoresponse is also observed in the fiber illuminated by optical pulses from a solar simulator (light intensity, 100 mW cm^−2^) with different bias voltages (Fig. 4D), illustrating its photoswitching behavior. The considerable photocurrent and stable dynamic photoresponse of the single-crystal organometallic perovskite fiber indicate its potential applications for in-fiber photodetection.

### Flexibility test

**Fig. 5.**
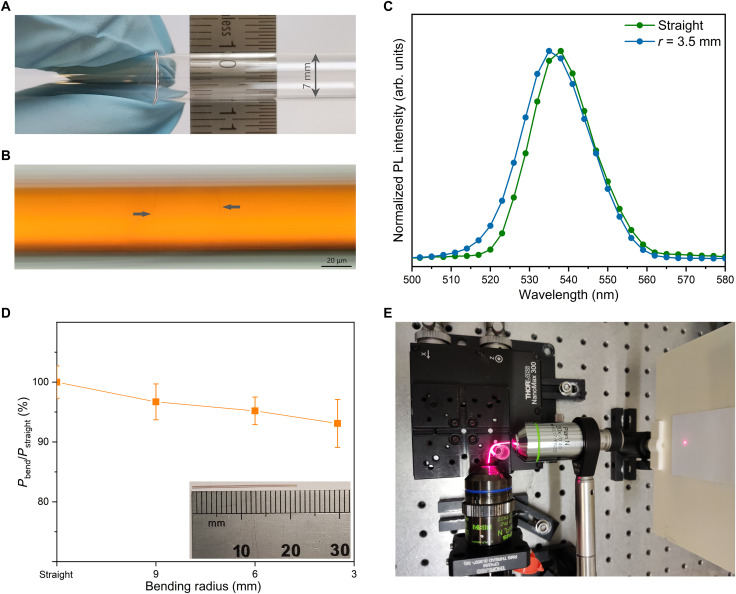
Flexibility test of the single-crystal MAPbBr_3_ optical fibers with PTFE cladding. (**A**) Photo of a 50-μm-core-diameter single-crystal MAPbBr_3_ perovskite optical fiber wrapped around a small tube (7 mm in diameter) to show its flexibility. (**B**) Optical microscopy image of the microcracks observed in the 50-μm-core-diameter MAPbBr_3_ perovskite optical fiber when the bending radius reaches about 3 mm. (**C**) Normalized confocal PL spectra of the 50-μm-core-diameter MAPbBr_3_ perovskite optical fiber with the bending radius of ∞ and 3.5 mm. The organometallic perovskite fiber was wrapped around on a small tube with a diameter of 7 mm for the PL measurement, and the measured bending tip should bear a tensile strain. (**D**) The change in output power of a 50-μm-core-diameter, 21-mm-long MAPbBr_3_ fiber as a function of the bending radius under the illumination of a 633-nm laser. Bottom right inset: the 50-μm-core-diameter, 21-mm-long MAPbBr_3_ fiber used for the test. (**E**) Photo of 633-nm light transmission in the 50-μm MAPbBr_3_ optical fiber with a bending radius of 3.5 mm.

The flexibility of a single-crystal organometallic perovskite optical fiber can be tailored by controlling its diameter. For a general material

$$\sigma_{\text{st}}\propto \frac{\mathrm{Eh}}{2r}$$

where σ_st_ is the maximum stress of the material, *E* is the Young’s modulus, *h* is the thickness (diameter in our case), and *r* is the bending radius (*36*). Reducing fiber diameter is beneficial to flexibility. To measure the minimal bending radius, the organometallic perovskite fibers with PTFE cladding were wrapped around cylinders or cones with various diameters. As shown in Fig. 5A, the 50-μm-core-diameter MAPbBr_3_ perovskite optical fiber with PTFE cladding can be wrapped around a small tube (7 mm in diameter) without any observable microcrack, demonstrating good flexibility. The minimal bending radii are 3.5 and 15 mm for the 50- and 125-μm-core-diameter MAPbBr_3_ perovskite optical fibers, respectively. The minimal bending radius of our 50-μm fiber is comparable with 2-μm-thick single-crystal organometallic perovskite thin films (*36*) and is suitably small for most applications of such fibers. Figure 5B illustrates the microcracks observed in a 50-μm-core-diameter MAPbBr_3_ perovskite optical fiber when the bending radius reaches about 3 mm. Figure 5C illustrates the normalized confocal PL spectra of the 50-μm MAPbBr_3_ fiber with a bending radius of ∞ and 3.5 mm; a blue shift (3 nm) of the PL peak is observed on the bending tip of the fiber, due to the increased bandgap of this material under tensile strain (*50*). There have been several methods to introduce strain in organometallic perovskite materials, including mechanical deformation, lattice mismatch, thermal expansion mismatch, and phase transitions (*51*–*53*). Mechanical deformation should be the most convenient way to apply and control strains in organometallic perovskites. Our flexible single-crystal fiber provides a suitable structure that is convenient for mechanical deformation-induced strains to be generated. To the best of our knowledge, this is the first reported strain-induced PL shift of organometallic halide perovskites caused by mechanical deformation. This mechanical deformation–induced PL shift behavior of our organometallic perovskite fiber may suggest its possible applications as a stress sensor and wavelength-tunable light source. The light transmission property of our single-crystal organometallic perovskite optical fiber under different bending radii is studied. The output power shows a small decrease with the reduction of bending radius (Fig. 5D), which indicates a small bending loss of the 50-μm fiber even at 3.5-mm bending radius. This could be attributed to the good flexibility and large refractive index of the MAPbBr_3_ core (*54*). Light transmission photos in the tested fiber with different bending radii under the illumination of a 633-nm laser are shown in Fig. 5E and fig. S9.

### Multiphoton absorption properties

**Fig. 6.**
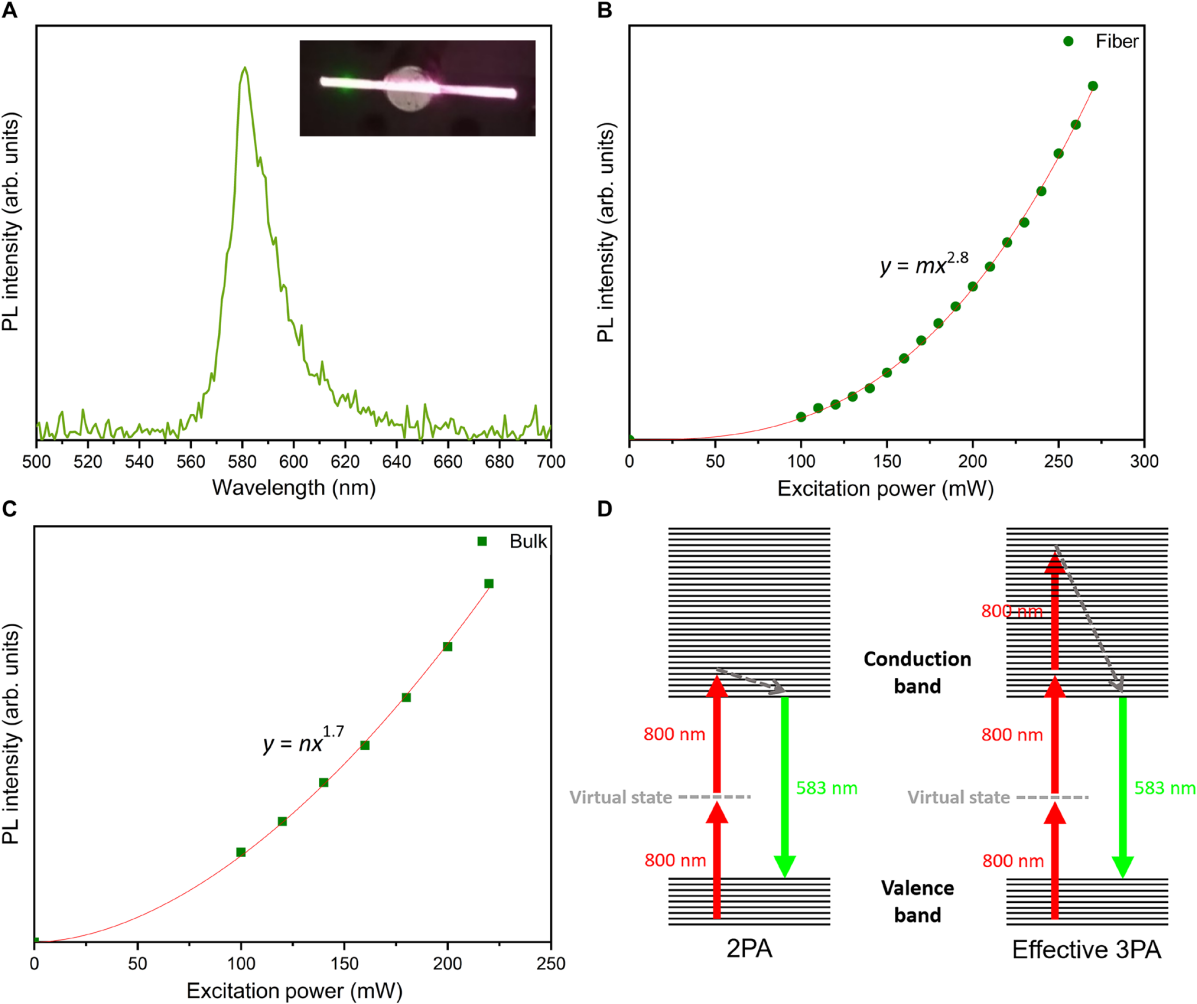
MPA properties of the single-crystal MAPbBr_3_ optical fiber. (**A**) Multiphoton-induced PL spectrum of the 500-μm-core-diameter single-crystal MAPbBr_3_ optical fiber. Inset: MPPL appears as the emission of green light in a 500-μm-core-diameter MAPbBr_3_ optical fiber. (**B**) Variation of the three-photon–induced PL intensity versus the incident laser power in a 500-μm-core-diameter MAPbBr_3_ optical fiber. (**C**) Variation of the two-photon–induced PL intensity versus the incident laser power in a common MAPbBr_3_ bulk single crystal. (**D**) The schematic diagrams of the possible processes of 2PA in single-crystal MAPbBr_3_ bulks and the effective 3PA in MAPbBr_3_ optical fibers.

Last, we study the multiphoton absorption (MPA) properties of single-crystal MAPbBr_3_ perovskite optical fibers. Nonlinear-transmission experiments are performed on the 500-μm-core-diameter fibers using 800-nm femtosecond laser pulses [central wavelength, 798 nm; full width at half maximum (FWHM), 113 nm; pulse duration, ~50 fs; and repetition rate, 80 MHz]. MPA is a high-order nonlinear process that takes place at high photon fluences. Two or more photons with energy less than the material’s bandgap are simultaneously absorbed and excite a charge carrier, which leads to the subsequent emission of a single photon at a higher energy. The inset of Fig. 6A shows that multiphoton-induced PL (MPPL) leads to green-light emission in a 500-μm-core-diameter MAPbBr_3_ optical fiber. Figure 6A illustrates the MPPL spectrum of a 500-μm-core-diameter single-crystal MAPbBr_3_ optical fiber. The MPPL spectra of most samples are centered around 583 nm (Fig. 6A), while one sample shows a blue shift of its spectrum (fig. S10). Although green-colored PL is observed (inset of Fig. 6A), the MPPL spectrum in Fig. 6A has a peak at 583 nm. This is mainly due to the self-absorption of higher-energy photons as the emitted light travels through the MAPbBr_3_ fibers, according to the steady-state absorbance curve in Fig. 4A.

The integrated MPPL intensity as a function of excitation power in 500-μm-core-diameter MAPbBr_3_ optical fibers was studied further. MAPbBr_3_ perovskite bulk single crystals were tested under the same experimental conditions for comparison. Figure 6 (B and C) shows the power dependence of MPPL intensity in a MAPbBr_3_ optical fiber and a MAPbBr_3_ bulk single crystal, respectively. The MPPL intensity shows a cubic dependence versus excitation power in all organometallic perovskite fibers, while a quadratic dependence is observed in the bulk single-crystal MAPbBr_3_. These results suggest that three-photon absorption (3PA) is dominant in organometallic perovskite optical fibers, while 2PA mainly happens in bulk single crystal. Although the reason is not entirely clear, here, we attempt to interpret the different nonlinear phenomena between the organometallic perovskite optical fiber and the bulk crystal. The 3PA of MAPbBr_3_ at 800 nm has never been reported before, as this material shows high absorbance for wavelengths shorter than ~550 nm and may consequently absorb high-intensity 800-nm light through 2PA directly. However, the absorbance of MAPbBr3 is even higher at shorter wavelengths (fig. S8), which may lead to 3PA with sufficient photon fluence at 800 nm (*55*). Similar to the simultaneous observations of 2PA and 3PA in some other semiconductors (*55*–*58*), the nonlinear absorption in organometallic perovskite optical fibers could belong to the effective 3PA, namely, two-photon–assisted excited free-carrier absorption, which is different from the standard instantaneous 3PA with the simultaneous absorption of three photons. The conditions necessary for effective 3PA are a sufficiently high excitation intensity and a longer excited-state lifetime than the pulse duration (*56*, *58*, *59*). Our single-crystal MAPbBr3 optical fibers may meet these conditions. First, the guidance and confinement of light in the perovskite fiber waveguide lead to a local excitation intensity higher than that in the bulk crystal to reach the threshold for effective 3PA at 800 nm. Second, the 50-fs pulse duration of the excitation laser is much smaller than the nanosecond-scale lifetime of the excited carriers in MAPbBr3 single crystals (*13*), which meets the second condition of longer excited-state lifetime. The mechanisms of 2PA in single-crystal MAPbBr3 bulks and effective 3PA in MAPbBr3 optical fibers are illustrated in Fig. 6D. The 2PA process is a dominant mechanism in the nonlinear absorption process in bulk single-crystal MAPbBr3 due to the relatively low excitation intensity. For the effective 3PA in MAPbBr_3_ optical fibers, the electrons in the valence band absorb two photons and make transitions to the conduction band. When the two-photon–generated free-carrier density exceeds a critical value due to the high excitation intensity, free-carrier absorption occurs (*60*). Then, the electrons in the conduction band can absorb additional photons, simultaneously generating transient intraband transitions to higher excited states, which form an equivalent stepwise (2PA:1PA) 3PA process, as shown in Fig. 6D (*56*, *58*). Subsequently, these electrons relax exceedingly fast to the bottom of the conduction band via a nonradiative transition. Last, electrons at the bottom of the conduction band relax to the valence band, recombining with holes resulting in PL. Therefore, two-photon–assisted excited free-carrier absorption due to the higher photon fluence in the MAPbBr_3_ optical fibers could be the predominant mechanism of the effective 3PA process, resulting in the different MPA properties compared with the bulk crystal counterpart under the same experimental conditions.

## DISCUSSION

In summary, we have demonstrated a solution-processed and scalable method for one-directional single-crystal organometallic perovskite growth, resulting in an organometallic perovskite optical fiber based on this. Our method is universal and can be extended to other types of organometallic perovskite materials. The single-crystal organometallic perovskite optical fibers exhibit low transmission loss and good stability under ambient conditions. Our 50-μm-core-diameter fibers with soft PTFE cladding are mechanically flexible, and the low transmission power decrease after bending indicates a low bending loss of the 50-μm fiber even at 3.5-mm bending radius. These flexible fibers are convenient for mechanical deformation–induced strains to be generated, leading to observable mechanical deformation–induced PL shift in organometallic perovskites. Unlike 2PA in bulk organometallic perovskite single crystals, up-conversion PL through 3PA is generated and guided in our single-crystal organometallic perovskite optical fibers under the same experimental conditions. The single-crystal organometallic perovskite optical fibers reported in this work may find applications in the fields of fiber optics and optoelectronics, such as optical modulation, up-conversion PL, fiber photodetector, and sensing.

## MATERIALS AND METHODS

### Chemicals and reagents

Lead bromide (98%), lead chloride (98%), dimethyl sulfoxide (DMSO; anhydrous, 99.9%), and *N*,*N*′-dimethylformamide (DMF; anhydrous, 99.8%) were purchased from Sigma-Aldrich. MABr and MACl were purchased from Ossila. All chemicals were used as received without any further purification.

### Synthesis of single-crystal organometallic perovskite optical fibers

For the synthesis of single-crystal MAPbBr_3_ fibers, PbBr_2_ and MABr (1/1 by molar, 1 M) were dissolved in DMF by magnetic stirring at room temperature. The solution was filtered using a PTFE filter with 0.2-μm pore size. The organometallic perovskite precursor solution was filled into the silica capillary tubes and PTFE capillary tubes of different inner diameters (50, 100, 125, 300, 500, and 1000 μm). The capillary tubes with precursor solution were sealed at one end to prevent leakage. Then, the capillary tube was fixed to ensure that the tube axis is perpendicular to the ground with the sealed end down, as shown in Fig. 1A. A heat-transfer block was placed on the hot plate and had a line contact with the capillary. The length of liquid column in the capillary that contacts with the block was controlled to a couple of millimeters at the beginning to prevent disordered nucleation. This line contact in combination with the limited heating region prevented disordered nucleation in other sites. At first, the solution at the bottom end was heated to 80°C and after a few hours, the seed organometallic perovskite crystal was formed. Then, the hot-plate temperature was reduced and kept at 60°C for the single-crystal fiber growth. In addition, extra heat-transfer blocks were added on top of each other according to the growth speed of the single-crystal organometallic perovskite optical fiber to ensure that the top block was always located at the crystal-solution interface, and therefore, a constant-temperature gradient was maintained in front of the crystal-solution interface by the changed heating position. This temperature gradient was essential, as it not only provided necessary ion diffusion for the continuous growth of organometallic perovskite core due to the thermal convection in the solution but also inhibited other nucleation sites because of the unsaturation at relative lower-temperature zones outside the crystal-solution interface. The precursor solution was refreshed periodically during the crystal growth to produce longer organometallic perovskite optical fiber. A solution container can be attached to the top end of the capillary tube for the convenience of solution renewal. Eventually, the organometallic perovskite optical fiber of a certain length was formed with a single-crystal organometallic perovskite core and a cladding. The growth rates are about 4 and 1.5 mm/day for the single-crystal MAPbBr_3_ perovskite optical fibers (glass cladding) with inner diameters of 500 and 300 μm, respectively. The precursor solution should be refreshed when the growth rate becomes lower than normal value. The growth rate of the 100-μm fiber (glass cladding) is about 0.5 mm/day at the very beginning and decreases gradually during the growth process until it finally stopped (the lengths that we currently can achieve are limited to several millimeters). The growth rate is about 0.5 to 0.7 mm/day for the MAPbBr_3_ perovskite fibers (PTFE cladding) with inner diameters of 50 and 125 μm. For the synthesis of single-crystal MAPbBr_2.5_Cl_0.5_ fibers, MABr (0.112 g), PbBr_2_ (0.275 g), and PbCl_2_ (0.070 g) were dissolved in DMF (1 ml) as the precursor solution. The temperatures for nucleation and crystal growth are 70° and 50°C, respectively. For the synthesis of single-crystal MAPbCl_3_ fibers, PbCl_2_ and MACl (1/1 by molar, 1 M) were dissolved in DMF-DMSO (1:1 v/v) as the precursor solution. The temperatures for nucleation and crystal growth are 50° and 40°C, respectively. To grow much longer fibers, an alternative fabrication approach could be used, as shown in fig. S11.

### Material characterization

Optical images were captured using an Olympus BX60 upright compound microscope. The SEM images were obtained using FEI Nova NanoSEM 650, operating in low-vacuum mode with an accelerating voltage of 5 kV. The chemical composition was determined by means of EDX (attached to FEI Nova NanoSEM 650) at a voltage of 10 kV. X-ray powder diffraction data were collected using a Bruker D8 Advance diffractometer in Bragg-Brentano geometry and operating with Ni-filtered Cu Kα radiation (λ = 1.5418 Å) over the 2θ range from 5° to 70°. Raman spectroscopies were obtained using a Renishaw inVia confocal Raman microscope at an excitation wavelength of 633 nm to avoid the fluorescence background. The synchrotron XRD measurements were undertaken using the beam line I18 at the Diamond Light Source, based at Didcot Oxfordshire (UK). The instrument was operated in the transmission model at 13 keV with a beam spot diameter of ≈25 μm (300-μm-core-diameter fiber) and 2.5 μm (50-μm-core-diameter fiber). About the 300-μm fiber, the Bragg-diffracted x-rays were measured at 25- and 100-μm intervals along the width and length of the fiber, respectively. As for the 50-μm fiber, the x-rays were measured at 10- and 250-μm intervals along the width and length. The steady-state absorption and photoluminescence were recorded using Perkin Elmer Lambda 950 UV-vis spectrophotometer with an integrating sphere and Perkin Elmer LS55 spectrofluorometer, respectively.

### Optical loss measurements

A 785-nm laser (LML-785.0CB-03 Laser from PD-LD Inc.) was used for loss measurements using the cutback method. Before the loss measurement, the fiber was polished using normal procedures without water. Light was coupled into the fiber in free space using a 40×/0.75–numerical aperture (NA) objective lens and the transmitted light was collected onto the Thorlabs PM110D power meter with a photodiode sensor and the output image was captured by a CCD camera (uEye UI-2230-C) with a 20× objective lens. The fiber output end was sequentially removed by 3 mm several times via cutting and polishing during the cutback measurements.

### Photodetector fabrication and measurements

For photodetector fabrication, the two ends of a 500-μm-core-diameter, 1-cm-long MAPbBr_3_ fiber were coated by silver conductive paint. After the silver paint dried up, conductive copper tapes were attached to both ends of the fiber and fixed by the silver paint. The *I*-*V* curves and dynamic photo response of the fiber photodetector were measured using Keithley 2400 under illumination of a 532-nm laser diode and solar simulator, respectively.

### Optical transmission measurements of flexible fiber

The fiber was cut by sharp blades and used for the measurements directly. A 633-nm laser was coupled into the fiber in free space using a 60×/0.70-NA objective lens, and the transmitted light was collected onto the Thorlabs PM110D power meter with a photodiode sensor.

### Confocal PL spectra measurements

Confocal PL spectra of flexible organometallic perovskite fibers were measured by a Zeiss LSM 710 confocal microscope at an excitation wavelength of 405 nm. The fibers were wrapped around on a small tube to measure the PL of the fibers under bending state.

### Nonlinear-transmission experiments

To study the MPA properties of MAPbBr_3_ optical fibers, a Ti:sapphire laser (brand, Coherent Micra; central wavelength, 798 nm; FWHM, 113 nm; pulse width, ~50 fs; repetition rate, 80 MHz; beam diameter, 0.42 mm) was used to pump the organometallic perovskite optical fibers. The fibers were mounted on a precision stage for alignment along three axes. The light was focused tightly using a long–focal length lens (150 mm). The fibers were then aligned with the laser beam. The output light from the fibers was collected by a large-NA objective and focused by a second lens onto a fiber coupler to an Ocean Optics spectrometer. A reflective filter for the 800-nm light was placed in between the lens and the fiber coupler to remove the majority of the pump beam.

## Supplementary Materials

### This PDF file includes:

Supplementary Text Figs. S1 to S11 Table S1 References
