# Supplementary material for: Single-crystal organometallic perovskite optical fibers
Source: Sci Adv. 2022 Sep 23;8(38):eabq8629. doi: 10.1126/sciadv.abq8629 (PMC9506722; doi:10.1126/sciadv.abq8629)
Supplement: Supplementary file 1 — Supplementary Text Figs. S1 to S11 Table S1 References [file sciadv.abq8629_sm.pdf]

Science Advances
AAAS

Supplementary Materials for

Single-crystal organometallic perovskite optical fibers

Yongfeng Zhou *et al.*

Corresponding author: Lei Su, l.su@qmul.ac.uk

Sci. Adv. **8**, eabq8629 (2022)
DOI: 10.1126/sciadv.abq8629

This PDF file includes:

Supplementary Text
Figs. S1 to S11
Table S1
References

Supplementary Text

The influence of convective current in the growth of single-crystal organometallic perovskite optical fibers

Current main fabrication methods of single-crystal organometallic perovskites include antisolvent vapour-assisted crystallization (AVC), liquid diffused separation induced crystallization (LDSC), temperature lowering crystallization (TLC) and inverse temperature crystallization (ITC). AVC and LDSC are room temperature methods, which fabricate single-crystal organometallic perovskites with low-defect density. In comparison, TLC and ITC operate at relatively high temperatures, which usually results in more defects. In normal TLC and ITC, the solution is heated from the bottom uniformly, the horizontal thermal distribution in the solution usually does not change, while thermal gradients exist in the vertical direction. So, convective currents arising from thermal gradients only exist in the vertical direction of the precursor solution in normal TLC and ITC. This convective current usually disturbs the ordered growth, leading to undesired twining defects and cracks in single crystals (*13, 15*). Nevertheless, single crystals fabricated using TLC and ITC possess better crystal quality and have lower defect density than their polycrystalline counterparts (*16*).

In our case of single-crystal fiber growth, a horizontal thermal gradient exists in the precursor solution due to the line contact between the capillaries and the heat transfer block. As a result, the convective current should exist in both horizontal and vertical directions in the precursor solution in the capillary for single-crystal organometallic perovskite fiber growth. In the vertical direction, the convective current in our method is similar to that in the standard ITC method (i.e. driven by the buoyancy and surface tension together). In the horizontal direction, however, the convection should only be driven by the surface tension in our case. Usually, in liquid films with thicknesses on or less than the millimeter scale, the surface tension dominates in driving the convection instead of buoyancy (*61*). Since the inner diameters of our capillaries are on the micrometer scale, the convection in the horizontal direction follows this model. In addition, the density gradient, or the gravimetric gradient of the precursor solution caused by the thermal gradient in the horizontal direction cannot generate the buoyancy-driven convection in this direction because they are in the same horizontal plane. Consequently, there is only surface-tension-driven convective current in the horizontal direction. The surface tension of the precursor solution decreases monotonically with increasing temperature. Due to thermal gradients in the solution, a surface tension difference is established and drives the liquid convection from the point with low surface tension to a higher one. Surface-tension-driven convection is usually assessed by the dimensionless Marangoni number (M_a), which is expressed as (*61, 62*)

$$M_a = -\frac{d\gamma}{dT}\frac{\Delta T d}{\rho v k}$$

where γ is the surface tension, ρ is the density, v is the kinematic viscosity, k is the thermal diffusivity, ΔT is the temperature difference, and d is the convection distance (core diameter in our case). Although we cannot measure the temperature difference ΔT in the horizontal direction of the precursor solution, the temperature difference in this direction should be much smaller than that in the vertical direction, as the inner diameters of the capillaries are relatively small and the capillaries are inserted in the center of the heat transfer block during the crystal growth. Besides, the convection distance d is on a micrometer scale in the horizontal direction, whereas it is at least a few millimeters in the vertical direction. Therefore, the surface-tension-driven convection should be much smaller in the horizontal direction than that in the vertical direction. In addition, the convective current in the vertical direction should be driven by the buoyancy and surface tension

together, while there is only the surface-tension-driven convection in the horizontal direction. All these suggests that the convection in the horizontal direction is much smaller than that in the vertical direction. Furthermore, the growth temperature for our single-crystal MAPbBr3 fibers is 60 ℃. This temperature is smaller than the one (90 ℃) used in the ITC method reported (*15*). A lower temperature used in our method further reduces the convection in our precursor solution. To summarize, in our single-crystal-fiber growth method, the negative influence of the convection in the horizontal direction on the crystal quality is believed to be minimal, and the influence of convective current in the vertical direction is also less than that in standard ITC methods due to the lower growth temperature employed.

In general, the convection in the precursor solution during the crystallization introduces defects in the crystals. For single-crystal organometallic perovskites, the convective current may lead to twining defects and cracks in single crystals (*13, 15*). These are sub-grain defects present in single crystals. The presence of such defects should not affect the single-crystal nature of our fibers. To observe twining structures, the chemical etching method for bulk crystals (*63*) or the specialized and carefully established low-dose rapid acquisition TEM method for thin films (*64*) are required. However, these methods and the associated observation are difficult to be applied to our fibers due to the cladding and fiber shape.

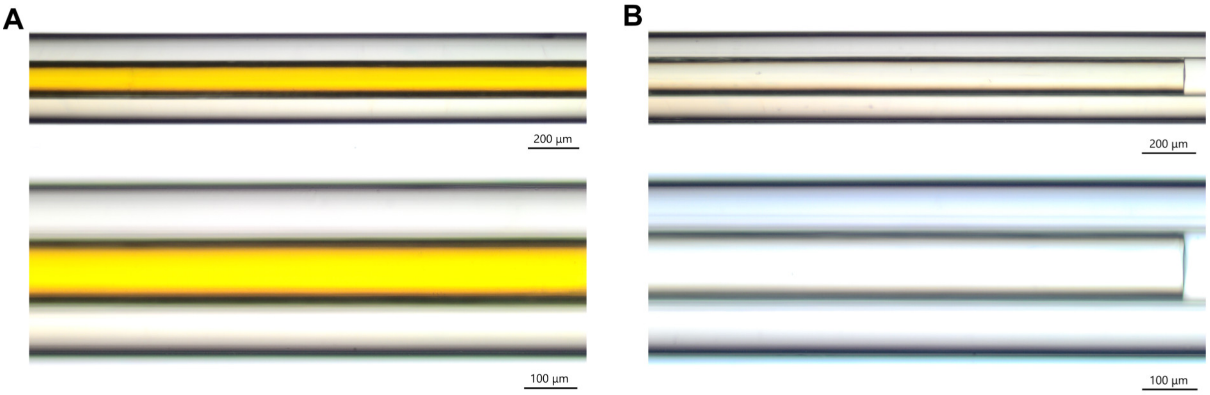

Fig. S1. Optical microscopy images of different single-crystal organometallic perovskite optical fibers. (A) Optical microscopy images of 125 µm core-diameter $MAPbBr_{2.5}Cl_{0.5}$ perovskite optical fibers. (B) Optical microscopy images of 125 µm core-diameter $MAPbCl_3$ perovskite optical fibers.

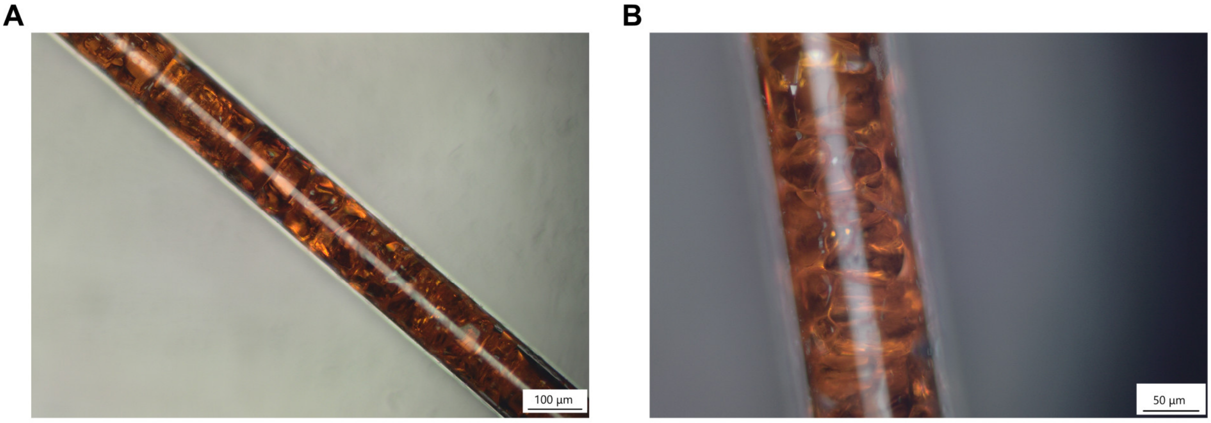

Fig. S2. Optical microscopy images of $MAPbBr_3$ polycrystalline fibers.

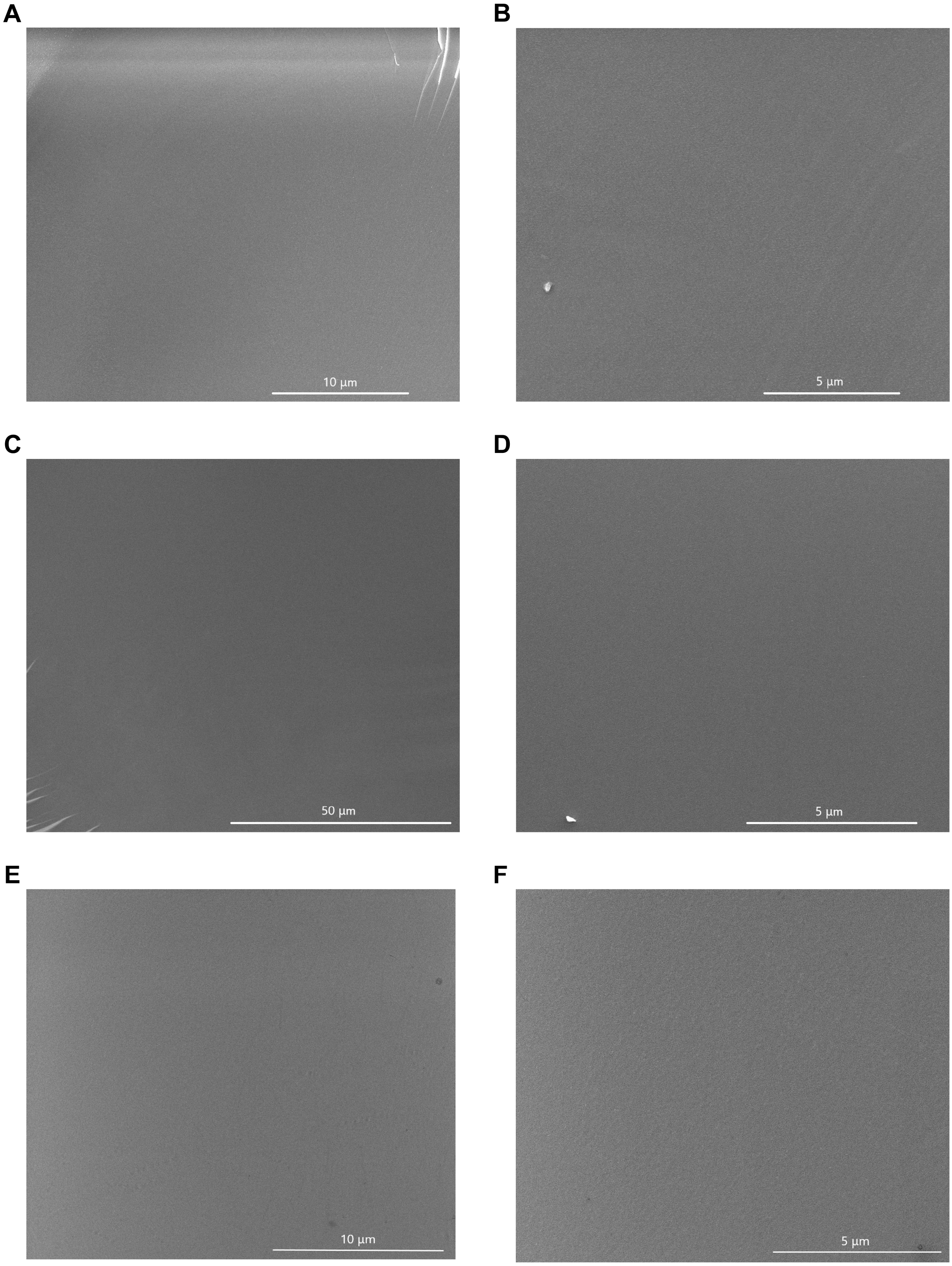

Fig. S3. High-resolution SEM images of the $MAPbBr_3$ fiber cross sections. (A, B) SEM images of the 300µm core-diameter fiber cross sections with silica cladding. (C, D) SEM images of the

500μm core-diameter fiber cross sections with silica cladding. (E, F) SEM images of the 50μm core-diameter fiber cross sections with PTFE cladding.

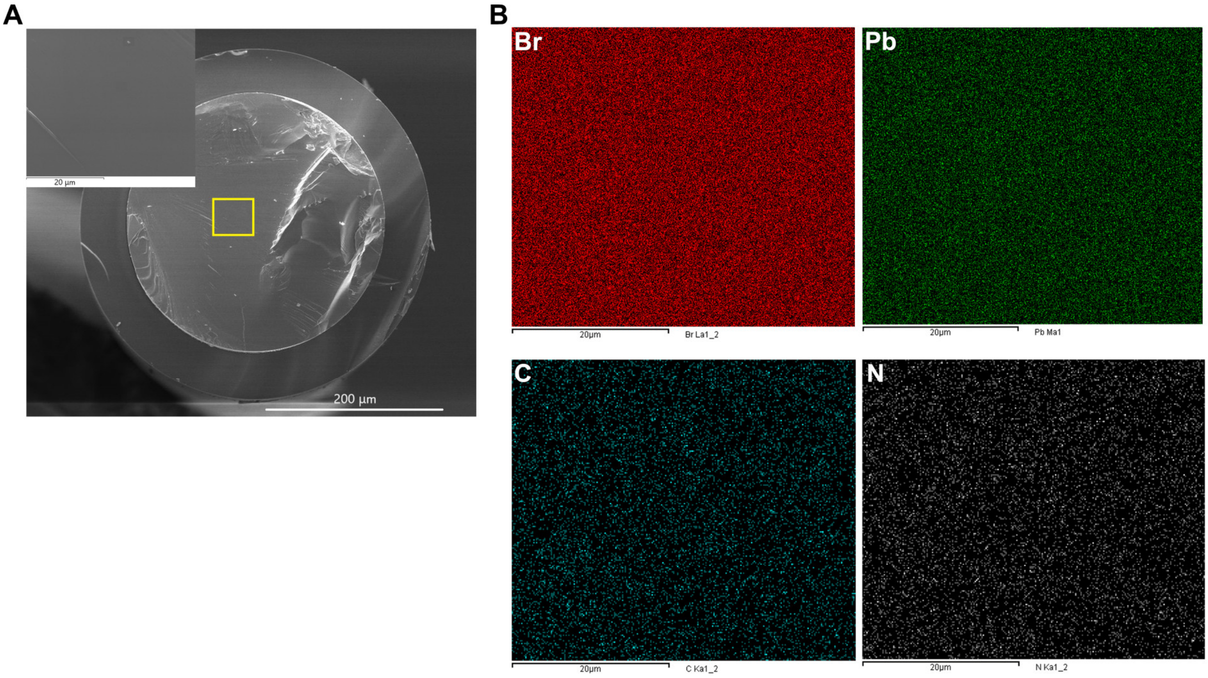

Fig. S4. EDX analysis of the $MAPbBr_3$ perovskite optical fiber. (A) SEM image of the fiber cross section with 300µm core-diameter. Inset: Enlarged image of the elements mapping zone. (B) Elemental mapping images for the $MAPbBr_3$ fiber.

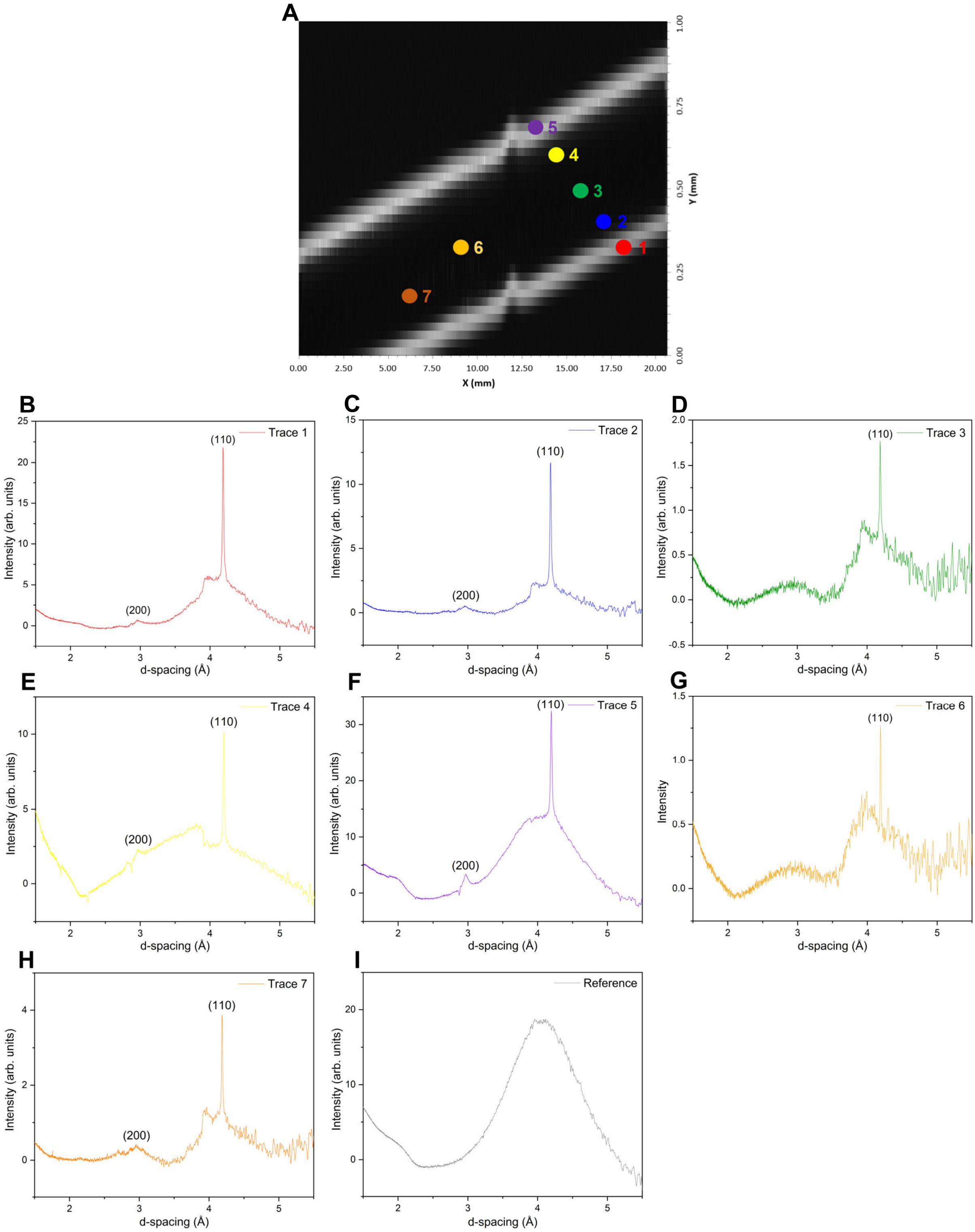

Fig. S5. Synchrotron XRD analysis of the single-crystal $MAPbBr_3$ optical fiber with 300 µm core diameter. (A) The intensity map of (110) peak in the whole organometallic perovskite fiber range. (B-H) d-spacing distributions and corresponding intensities on different positions in the

organometallic perovskite fiber. (I) Reference curve of the glass cladding without organometallic perovskite core.

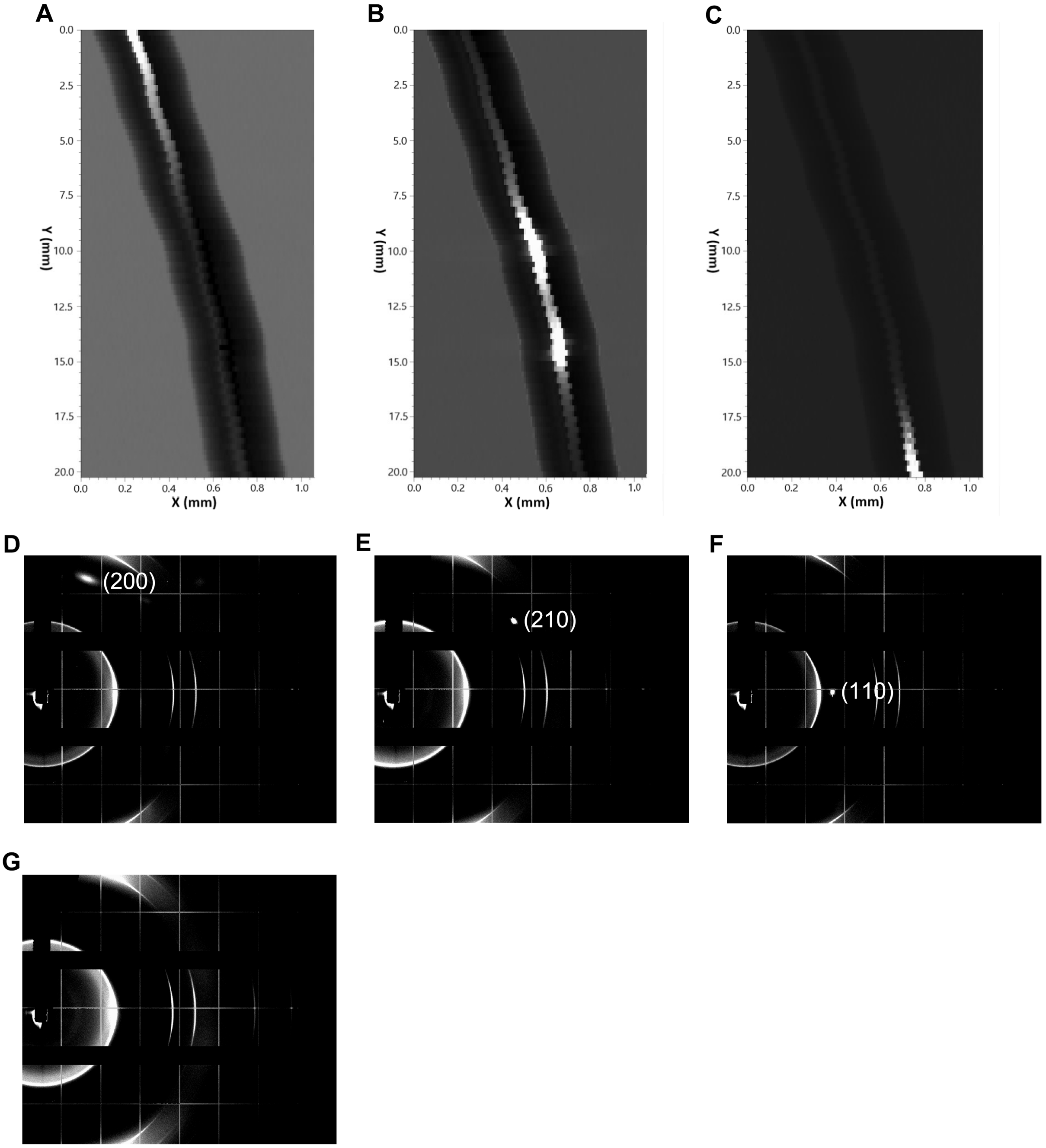

Fig. S6. Synchrotron XRD analysis of the flexible single-crystal $MAPbBr_3$ optical fiber with 50 µm core diameter. (A-C) The intensity map of the (200), (210) and (110) peak in the whole organometallic perovskite fiber range through synchrotron XRD analysis, respectively. A single (200) peak appears on the top of the fiber, and its intensity gradually reduces and disappears with the downward scan. In the region where (200) peak intensity starts to reduce, a (210) peak is observed, and its intensity slowly increases with the decrease of (200) peak intensity. Then, the (200) peak disappears and only the (210) peak can be observed in the fiber core. With the further downward scan, the (210) peak intensity also begins to reduce. Next, a (110) peak emerges when the (210) peak intensity reduces, and its intensity gradually rises with the decrease of (210) peak

intensity. Finally, the (210) peak fades away and only the (110) peak can be found near the bottom of fiber core. (D-F) Diffraction spot of (200), (210) and (110) in the single-crystal $MAPbBr_3$ fiber, respectively. (G) Reference diffraction pattern of the PTFE cladding without organometallic perovskite core.

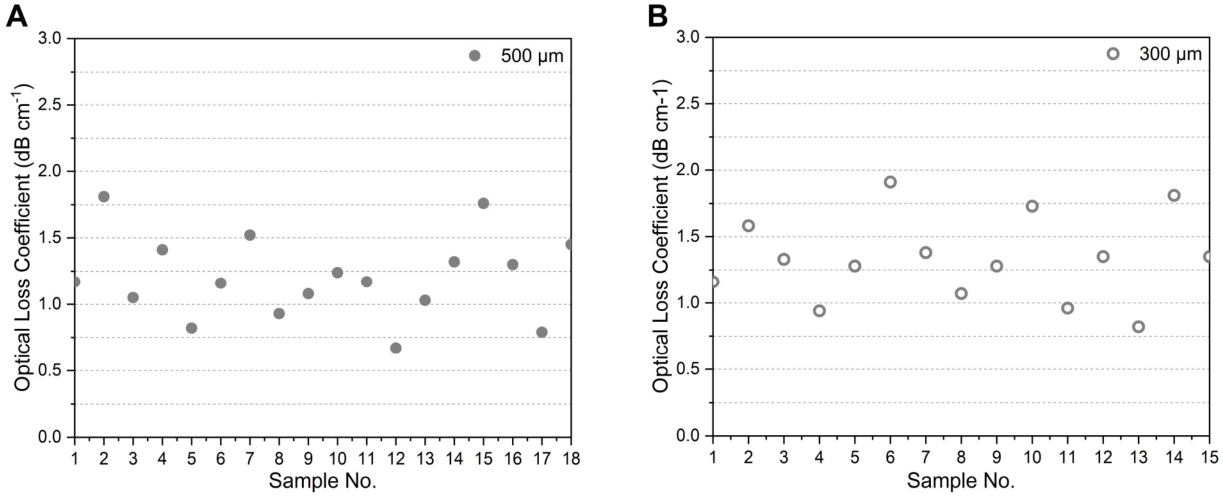

Fig. S7. The optical loss coefficients of single-crystal $MAPbBr_3$ optical fibers at wavelength 785 nm. (A) The optical loss coefficients of $MAPbBr_3$ optical fibers with 500μm core-diameter. (B) The optical loss coefficients of $MAPbBr_3$ optical fibers with 300μm core-diameter.

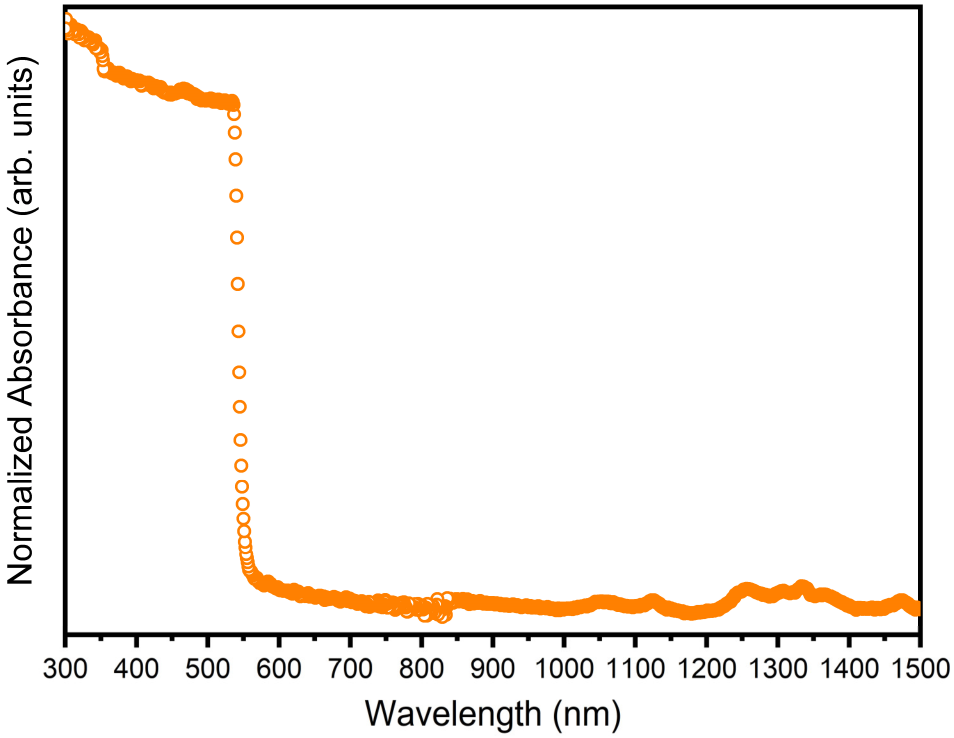

Fig. S8. Steady-state absorbance spectrum of 500 µm $MAPbBr_3$ optical fibers.

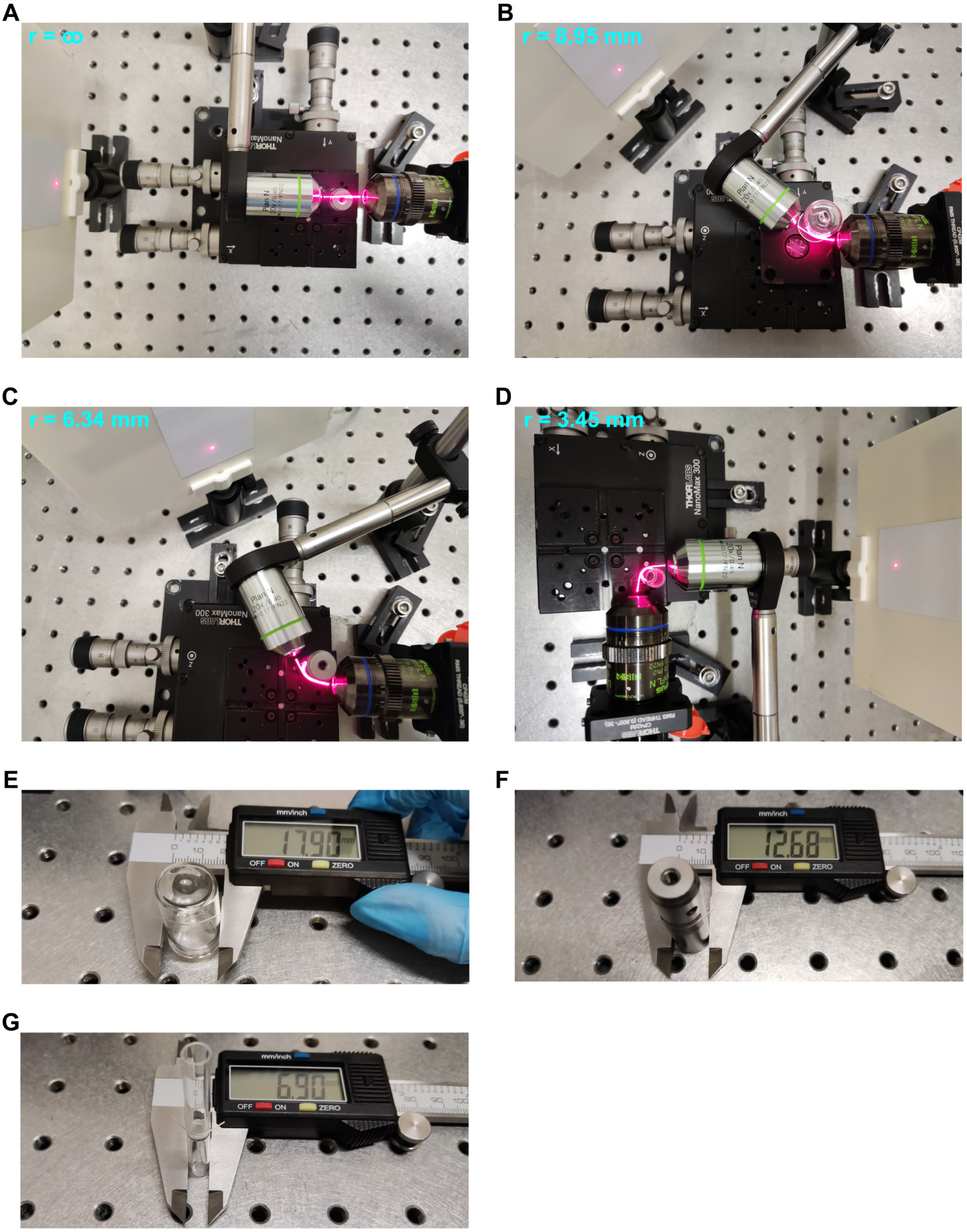

Fig. S9. Light transmission in the 50 μm core-diameter 21 mm-long $MAPbBr_3$ optical fiber with different bending radiuses under the illumination of 633 nm laser. (A-D) Photos of 633 nm light

transmission in the 50 μm $MAPbBr_3$ optical fiber with the bending radius of ∞, 9 mm, 6.3 mm and 3.5 mm. (E-G) Photos of the tested fiber wrapped around cylinders with different diameters.

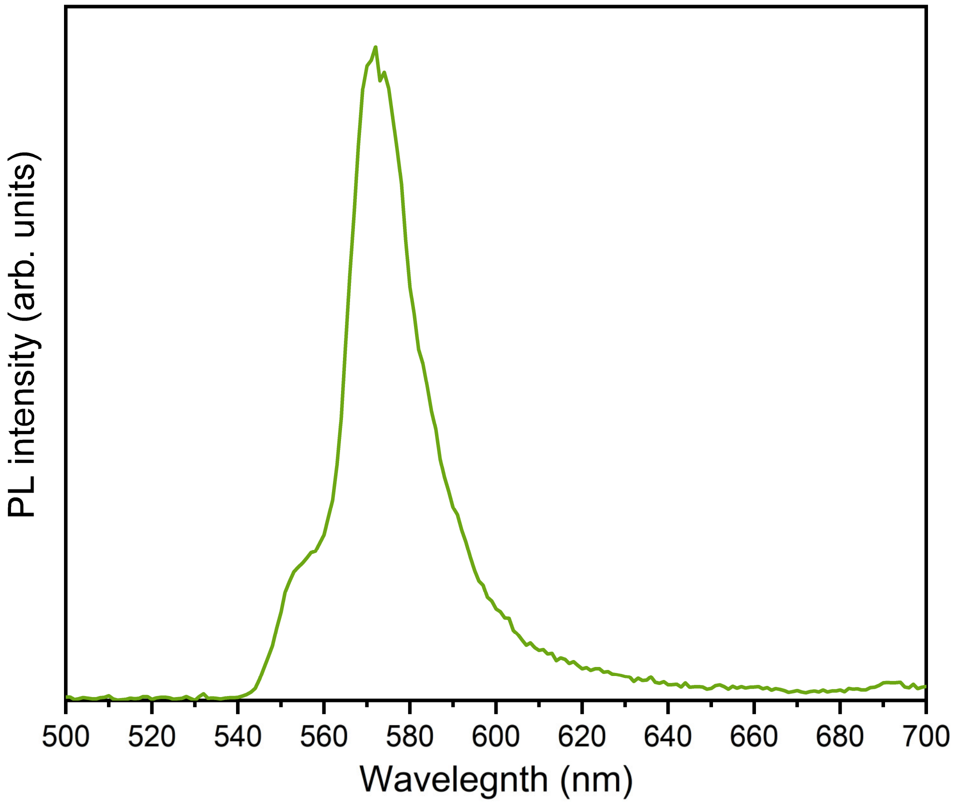

Fig. S10. The blue shift of three-photon-induced PL spectrum in a 500 µm core-diameter single-crystal $MAPbBr_3$ optical fiber.

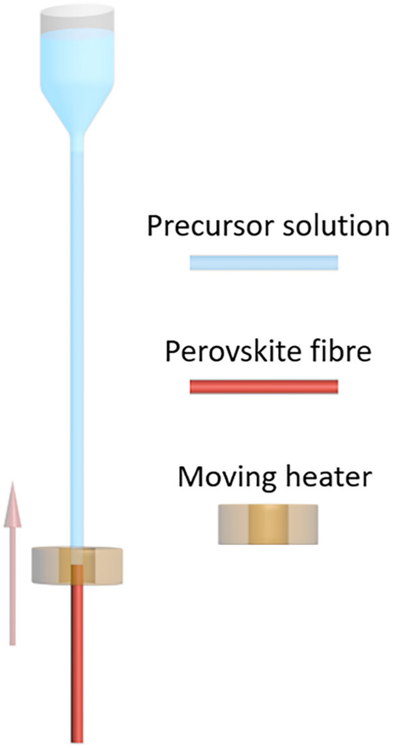

Fig. S11. The schematic diagram of a theoretical fabrication approach for much longer organometallic perovskite optical fibers.

Table 1. The measured Raman shift of vibration modes and peak assignments of $MAPbBr_3$ fibers

Raman shift (cm^{-1})	Peak assignment
325	$CH_3NH_3^+$ restricted rotation
915	$CH_3NH_3^+$ rocking
969	C-N stretching
1252	CH_3-NH_3^+ rocking
1479	Asym. NH_3^+ bending
1590	NH_3^+ twisting
2825	N^+-H stretching
2891	Asym. CH_3 stretching
2967	Sym. CH_3 stretching
3039	
3117	NH_3^+ Sym. stretching
3178	

REFERENCES AND NOTES

1. M. M. Lee, J. Teuscher, T. Miyasaka, T. N. Murakami, H. J. Snaith, Efficient hybrid solar cells based on meso-superstructured organometal halide perovskites. *Science* **338**, 643–647 (2012).

2. W. Li, Z. Wang, F. Deschler, S. Gao, R. H. Friend, A. K. Cheetham, Chemically diverse and multifunctional hybrid organic–inorganic perovskites. *Nat. Rev. Mater.* **2**, 16099 (2017).

3. C. Wehrenfennig, G. E. Eperon, M. B. Johnston, H. J. Snaith, L. M. Herz, High charge carrier mobilities and lifetimes in organolead trihalide perovskites. *Adv. Mater.* **26**, 1584–1589 (2014).

4. M. R. Filip, G. E. Eperon, H. J. Snaith, F. Giustino, Steric engineering of metal-halide perovskites with tunable optical band gaps. *Nat. Commun.* **5**, 5757 (2014).

5. N. J. Jeon, J. H. Noh, W. S. Yang, Y. C. Kim, S. Ryu, J. Seo, S. I. Seok, Compositional engineering of perovskite materials for high-performance solar cells. *Nature* **517**, 476–480 (2015).

6. Z.-K. Tan, R. S. Moghaddam, M. L. Lai, P. Docampo, R. Higler, F. Deschler, M. Price, A. Sadhanala, L. M. Pazos, D. Credgington, F. Hanusch, T. Bein, H. J. Snaith, R. H. Friend, Bright light-emitting diodes based on organometal halide perovskite. *Nat. Nanotechnol.* **9**, 687–692 (2014).

7. Z. Yang, Y. Deng, X. Zhang, S. Wang, H. Chen, S. Yang, J. Khurgin, N. X. Fang, X. Zhang, R. Ma, High-performance single-crystalline perovskite thin-film photodetector. *Adv. Mater.* **30**, 1704333 (2018).

8. H. Zhu, Y. Fu, F. Meng, X. Wu, Z. Gong, Q. Ding, M. V. Gustafsson, M. T. Trinh, S. Jin, X. Y. Zhu, Lead halide perovskite nanowire lasers with low lasing thresholds and high quality factors. *Nat. Mater.* **14**, 636–642 (2015).

9. T.-C. Wei, S. Mokkapati, T.-Y. Li, C.-H. Lin, G.-R. Lin, C. Jagadish, J.-H. He, Nonlinear absorption applications of $CH_3NH_3PbBr_3$ perovskite crystals. *Adv. Funct. Mater.* **28**, 1707175 (2018).

10. F. O. Saouma, C. C. Stoumpos, J. Wong, M. G. Kanatzidis, J. I. Jang, Selective enhancement of optical nonlinearity in two-dimensional organic-inorganic lead iodide perovskites. *Nat. Commun.* **8**, 742 (2017).

11. Y.-M. You, W.-Q. Liao, D. Zhao, H.-Y. Ye, Y. Zhang, Q. Zhou, X. Niu, J. Wang, P.-F. Li, D.-W. Fu, Z. Wang, S. Gao, K. Yang, J.-M. Liu, J. Li, Y. Yan, R.-G. Xiong, An organic-inorganic perovskite ferroelectric with large piezoelectric response. *Science* **357**, 306–309 (2017).

12. W. Tang, J. Zhang, S. Ratnasingham, F. Liscio, K. Chen, T. Liu, K. Wan, E. S. Galindez, E. Bilotti, M. Reece, M. Baxendale, S. Milita, M. A. McLachlan, L. Su, O. Fenwick, Substitutional doping of hybrid organic–inorganic perovskite crystals for thermoelectrics. *J. Mater. Chem. A* **8**, 13594–13599 (2020).

13. D. Shi, V. Adinolfi, R. Comin, M. Yuan, E. Alarousu, A. Buin, Y. Chen, S. Hoogland, A. Rothenberger, K. Katsiev, Y. Losovyj, X. Zhang, P. A. Dowben, O. F. Mohammed, E. H. Sargent, O. M. Bakr, Low trap-state density and long carrier diffusion in organolead trihalide perovskite single crystals. *Science* **347**, 519–522 (2015).

14. Y. Dang, Y. Liu, Y. Sun, D. Yuan, X. Liu, W. Lu, G. Liu, H. Xia, X. Tao, Bulk crystal growth of hybrid perovskite material $CH_3NH_3PbI_3$. *CrstEngComm* **17**, 665–670 (2015).

15. F. Yao, J. Peng, R. Li, W. Li, P. Gui, B. Li, C. Liu, C. Tao, Q. Lin, G. Fang, Room-temperature liquid diffused separation induced crystallization for high-quality perovskite single crystals. *Nat. Commun.* **11**, 1194 (2020).

16. M. I. Saidaminov, A. L. Abdelhady, B. Murali, E. Alarousu, V. M. Burlakov, W. Peng, I. Dursun, L. Wang, Y. He, G. Maculan, A. Goriely, T. Wu, O. F. Mohammed, O. M. Bakr, High-quality bulk hybrid perovskite single crystals within minutes by inverse temperature crystallization. *Nat. Commun.* **6**, 7586 (2015).

17. P. Wang, C. C. O'Mahony, T. Lee, R. Ismaeel, T. Hawkins, Y. Semenova, L. Bo, Q. Wu, C. McDonagh, G. Farrell, J. Ballato, G. Brambilla, Mid-infrared Raman sources using spontaneous Raman scattering in germanium core optical fibers. *Appl. Phys. Lett.* **102**, 011111 (2013).

18. Y. P. Huang, L. A. Wang, In-line silicon Schottky photodetectors on silicon cored fibers working in 1550 nm wavelength regimes. *Appl. Phys. Lett.* **106**, 191106 (2015).

19. P. Mehta, N. Healy, T. D. Day, J. V. Badding, A. C. Peacock, Ultrafast wavelength conversion via cross-phase modulation in hydrogenated amorphous silicon optical fibers. *Opt. Express* **20**, 26110–26116 (2012).

20. M. Bayindir, F. Sorin, A. F. Abouraddy, J. Viens, S. D. Hart, J. D. Joannopoulos, Y. Fink, Metal–insulator–semiconductor optoelectronic fibres. *Nature* **431**, 826–829 (2004).

21. F. A. Martinsen, B. K. Smeltzer, M. Nord, T. Hawkins, J. Ballato, U. J. Gibson, Silicon-core glass fibres as microwire radial-junction solar cells. *Sci. Rep.* **4**, 6283 (2015).

22. T. Zhang, K. Li, J. Zhang, M. Chen, Z. Wang, S. Ma, N. Zhang, L. Wei, High-performance, flexible, and ultralong crystalline thermoelectric fibers. *Nano Energy* **41**, 35–42 (2017).

23. S. Egusa, Z. Wang, N. Chocat, Z. M. Ruff, A. M. Stolyarov, D. Shemuly, F. Sorin, P. T. Rakich, J. D. Joannopoulos, Y. Fink, Multimaterial piezoelectric fibres. *Nat. Mater.* **9**, 643–648 (2010).

24. D. Homa, A. Cito, G. Pickrell, C. Hill, B. Scott, Silicon fiber with p-n junction. *Appl. Phys. Lett.* **105**, 122110 (2014).

25. J. Ballato, T. Hawkins, P. Foy, R. Stolen, B. Kokuoz, M. Ellison, C. McMillen, J. Reppert, A. M. Rao, M. Daw, S. Sharma, R. Shori, O. Stafsudd, R. R. Rice, D. R. Powers, Silicon optical fiber. *Opt. Express* **16**, 18675–18683 (2008).

26. P. J. A. Sazio, A. Amezcua-Correa, C. E. Finlayson, J. R. Hayes, T. J. Scheidemantel, N. F. Baril, B. R. Jackson, D.-J. Won, F. Zhang, E. R. Margine, V. Gopalan, V. H. Crespi, J. V. Badding, Microstructured optical fibers as high-pressure microfluidic reactors. *Science* **311**, 1583–1586 (2006).

27. J. Ballato, T. Hawkins, P. Foy, C. M. Millen, L. Burka, J. Reppert, R. Podila, A. M. Rao, R. R. Rice, Binary III-V semiconductor core optical fiber. *Opt. Express* **18**, 4972–4979 (2010).

28. J. R. Sparks, R. He, N. Healy, M. Krishnamurthi, A. C. Peacock, P. J. A. Sazio, V. Gopalan, J. V. Badding, Zinc selenide optical fibers. *Adv. Mater.* **23**, 1647–1651 (2011).

29. H. K. Tyagi, M. A. Schmidt, L. Prill Sempere, P. S. J. Russell, Optical properties of photonic crystal fiber with integral micron-sized Ge wire. *Opt. Express* **16**, 17227–17236 (2008).

30. N. Healy, M. Fokine, Y. Franz, T. Hawkins, M. Jones, J. Ballato, A. C. Peacock, U. J. Gibson, CO_2 laser-induced directional recrystallization to produce single crystal silicon-core optical fibers with low loss. *Adv. Opt. Mater.* **4**, 1004–1008 (2016).

31. X. Ji, S. Lei, S.-Y. Yu, H. Y. Cheng, W. Liu, N. Poilvert, Y. Xiong, I. Dabo, S. E. Mohney, J. V. Badding, V. Gopalan, Single-crystal silicon optical fiber by direct laser crystallization. *ACS Photonics* **4**, 85–92 (2017).

32. X. Ji, R. L. Page, S. Chaudhuri, W. Liu, S.-Y. Yu, S. E. Mohney, J. V. Badding, V. Gopalan, Single-crystal germanium core optoelectronic fibers. *Adv. Opt. Mater.* **5**, 1600592 (2017).

33. S. A. Veldhuis, P. P. Boix, N. Yantara, M. Li, T. C. Sum, N. Mathews, S. G. Mhaisalkar, Perovskite materials for light-emitting diodes and lasers. *Adv. Mater.* **28**, 6804–6834 (2016).

34. G. Xing, N. Mathews, S. S. Lim, N. Yantara, X. Liu, D. Sabba, M. Grätzel, S. Mhaisalkar, T. C. Sum, Low-temperature solution-processed wavelength-tunable perovskites for lasing. *Nat. Mater.* **13**, 476–480 (2014).

35. J. Zhang, Q. Guo, X. Li, C. Li, K. Wu, I. Abrahams, H. Yan, M. M. Knight, C. J. Humphreys, L. Su, Solution-processed epitaxial growth of arbitrary surface nanopatterns on hybrid perovskite monocrystalline thin films. *ACS Nano* **14**, 11029–11039 (2020).

36. Y. Lei, Y. Chen, R. Zhang, Y. Li, Q. Yan, S. Lee, Y. Yu, H. Tsai, W. Choi, K. Wang, Y. Luo, Y. Gu, X. Zheng, C. Wang, C. Wang, H. Hu, Y. Li, B. Qi, M. Lin, Z. Zhang, S. A. Dayeh, M. Pharr, D. P. Fenning, Y.-H. Lo, J. Luo, K. Yang, J. Yoo, W. Nie, S. Xu, A fabrication process for flexible single-crystal perovskite devices. *Nature* **583**, 790–795 (2020).

37. W. Mao, J. Zheng, Y. Zhang, A. S. R. Chesman, Q. Ou, J. Hicks, F. Li, Z. Wang, B. Graystone, T. D. M. Bell, M. U. Rothmann, N. W. Duffy, L. Spiccia, Y.-B. Cheng, Q. Bao, U. Bach, Controlled growth of monocrystalline organo-lead halide perovskite and its application in photonic devices. *Angew. Chem. Int. Ed.* **56**, 12486–12491 (2017).

38. Z. Wang, J. Liu, Z.-Q. Xu, Y. Xue, L. Jiang, J. Song, F. Huang, Y. Wang, Y. L. Zhong, Y. Zhang, Y.-B. Cheng, Q. Bao, Wavelength-tunable waveguides based on polycrystalline organic–inorganic perovskite microwires. *Nanoscale* **8**, 6258–6264 (2016).

39. P. Liu, X. He, J. Ren, Q. Liao, J. Yao, H. Fu, Organic–inorganic hybrid perovskite nanowire laser arrays. *ACS Nano* **11**, 5766–5773 (2017).

40. N. Kurahashi, H. Mizuno, F. Sasaki, H. Yanagi, Whispering gallery mode lasing from $CH_3NH_3PbBr_3$/PEO composites grown in a microcapillary. *J. Phys. Chem. C* **124**, 3242–3249 (2020).

41. Z. Chen, Q. Dong, Y. Liu, C. Bao, Y. Fang, Y. Lin, S. Tang, Q. Wang, X. Xiao, Y. Bai, Y. Deng, J. Huang, Thin single crystal perovskite solar cells to harvest below-bandgap light absorption. *Nat. Commun.* **8**, 1890 (2017).

42. C. Bi, Q. Wang, Y. Shao, Y. Yuan, Z. Xiao, J. Huang, Non-wetting surface-driven high-aspect-ratio crystalline grain growth for efficient hybrid perovskite solar cells. *Nat. Commun.* **6**, 7747 (2015).

43. C. Sendner, D. Horinek, L. Bocquet, R. R. Netz, Interfacial water at hydrophobic and hydrophilic surfaces: Slip, viscosity, and diffusion. *Langmuir* **25**, 10768–10781 (2009).

44. Y.-X. Chen, Q.-Q. Ge, Y. Shi, J. Liu, D.-J. Xue, J.-Y. Ma, J. Ding, H.-J. Yan, J.-S. Hu, L.-J. Wan, General space-confined on-substrate fabrication of thickness-adjustable hybrid perovskite single-crystalline thin films. *J. Am. Chem. Soc.* **138**, 16196–16199 (2016).

45. L.-Q. Xie, T.-Y. Zhang, L. Chen, N. Guo, Y. Wang, G.-K. Liu, J.-R. Wang, J.-Z. Zhou, J.-W. Yan, Y.-X. Zhao, B.-W. Mao, Z.-Q. Tian, Organic–inorganic interactions of single crystalline organolead halide perovskites studied by Raman spectroscopy. *Phys. Chem. Chem. Phys.* **18**, 18112–18118 (2016).

46. Y. Liu, Z. Yang, D. Cui, X. Ren, J. Sun, X. Liu, J. Zhang, Q. Wei, H. Fan, F. Yu, X. Zhang, C. Zhao, S. Liu, Two-inch-sized perovskite $CH_3NH_3PbX_3$ (X = Cl, Br, I) crystals: Growth and characterization. *Adv. Mater.* **27**, 5176–5183 (2015).

47. S. Amari, J.-M. Verilhac, E. Gros D'Aillon, A. Ibanez, J. Zaccaro, Optimization of the growth conditions for high quality $CH_3NH_3PbBr_3$ hybrid perovskite single crystals. *Cryst. Growth Des.* **20**, 1665–1672 (2020).

48. Z. Ni, C. Bao, Y. Liu, Q. Jiang, W.-Q. Wu, S. Chen, X. Dai, B. Chen, B. Hartweg, Z. Yu, Z. Holman, J. Huang, Resolving spatial and energetic distributions of trap states in metal halide perovskite solar cells. *Science* **367**, 1352–1358 (2020).

49. F. Chen, C. Xu, Q. Xu, Y. Zhu, F. Qin, W. Zhang, Z. Zhu, W. Liu, Z. Shi, Self-assembled growth of ultrastable $CH_3NH_3PbBr_3$ perovskite milliwires for photodetectors. *ACS Appl. Mater. Interfaces* **10**, 25763–25769 (2018).

50. C. Zhu, X. Niu, Y. Fu, N. Li, C. Hu, Y. Chen, X. He, G. Na, P. Liu, H. Zai, Y. Ge, Y. Lu, X. Ke, Y. Bai, S. Yang, P. Chen, Y. Li, M. Sui, L. Zhang, H. Zhou, Q. Chen, Strain engineering in perovskite solar cells and its impacts on carrier dynamics. *Nat. Commun.* **10**, 815 (2019).

51. Y. Chen, Y. Lei, Y. Li, Y. Yu, J. Cai, M.-H. Chiu, R. Rao, Y. Gu, C. Wang, W. Choi, H. Hu, C. Wang, Y. Li, J. Song, J. Zhang, B. Qi, M. Lin, Z. Zhang, A. E. Islam, B. Maruyama, S. Dayeh, L.-J. Li, K. Yang, Y.-H. Lo, S. Xu, Strain engineering and epitaxial stabilization of halide perovskites. *Nature* **577**, 209–215 (2020).

52. D.-J. Xue, Y. Hou, S.-C. Liu, M. Wei, B. Chen, Z. Huang, Z. Li, B. Sun, A. H. Proppe, Y. Dong, M. I. Saidaminov, S. O. Kelley, J.-S. Hu, E. H. Sargent, Regulating strain in perovskite thin films through charge-transport layers. *Nat. Commun.* **11**, 1514 (2020).

53. D. Liu, D. Luo, A. N. Iqbal, K. W. P. Orr, T. A. S. Doherty, Z.-H. Lu, S. D. Stranks, W. Zhang, Strain analysis and engineering in halide perovskite photovoltaics. *Nat. Mater.* **20**, 1337–1346 (2021).

54. A. M. A. Leguy, P. Azarhoosh, M. I. Alonso, M. Campoy-Quiles, O. J. Weber, J. Yao, D. Bryant, M. T. Weller, J. Nelson, A. Walsh, M. van Schilfgaarde, P. R. F. Barnes, Experimental and

theoretical optical properties of methylammonium lead halide perovskites. *Nanoscale* **8**, 6317–6327 (2016).

55. T. Sarma, P. K. Panda, P. T. Anusha, S. V. Rao, Dinaphthoporphycenes: Synthesis and nonlinear optical studies. *Org. Lett.* **13**, 188–191 (2011).

56. A. A. Said, M. Sheik-Bahae, D. J. Hagan, T. H. Wei, J. Wang, J. Young, E. W. Van Stryland, Determination of bound-electronic and free-carrier nonlinearities in ZnSe, GaAs, CdTe, and ZnTe. *J. Opt. Soc. Am. B* **9**, 405–414 (1992).

57. P. A. Kurian, C. Vijayan, C. S. S. Sandeep, R. Philip, K. Sathiyamoorthy, Two-photon-assisted excited state absorption in nanocomposite films of PbS stabilized in a synthetic glue matrix. *Nanotechnology* **18**, 075708 (2007).

58. F. Wu, G. Zhang, W. Tian, W. Chen, G. Zhao, S. Cao, W. Xie, Two-photon absorption and two-photon assisted excited-state absorption in $CdSe_{0.3}S_{0.7}$ quantum dots. *J. Opt. A Pure Appl. Opt.* **11**, 065206 (2009).

59. B. Gu, K. Lou, H.-T. Wang, W. Ji, Dynamics of two-photon-induced three-photon absorption in nanosecond, picosecond, and femtosecond regimes. *Opt. Lett.* **35**, 417–419 (2010).

60. A. Cutolo, M. Iodice, P. Spirito, L. Zeni, Silicon electro-optic modulator based on a three terminal device integrated in a low-loss single-mode SOI waveguide. *J. Lightwave Technol.* **15**, 505–518 (1997).

61. F. Ye, W. Tang, F. Xie, M. Yin, J. He, Y. Wang, H. Chen, Y. Qiang, X. Yang, L. Han, Low-temperature soft-cover deposition of uniform large-scale perovskite films for high-performance solar cells. *Adv. Mater.* **29**, 1701440 (2017).

62. E. L. Koschmieder, M. I. Biggerstaff, Onset of surface-tension-driven Bénard convection. *J. Fluid Mech.* **167**, 49–64 (1986).

63. Y. Rakita, O. Bar-Elli, E. Meirzadeh, H. Kaslasi, Y. Peleg, G. Hodes, I. Lubomirsky, D. Oron, D. Ehre, D. Cahen, Tetragonal $CH_3NH_3PbI_3$ is ferroelectric. *Proc. Natl. Acad. Sci. U.S.A.* **114**, E5504–E5512 (2017).

64. M. U. Rothmann, W. Li, Y. Zhu, U. Bach, L. Spiccia, J. Etheridge, Y. Cheng, Direct observation of intrinsic twin domains in tetragonal $CH_3NH_3PbI_3$. *Nat. Commun.* **8**, 14547 (2017).
